# Fast collective motions of backbone in transmembrane α helices are critical to water transfer of aquaporin

**DOI:** 10.1126/sciadv.ade9520

**Published:** 2024-05-08

**Authors:** Huan Tan, Mojie Duan, Huayong Xie, Yongxiang Zhao, Hui Liu, Minghui Yang, Maili Liu, Jun Yang

**Affiliations:** ^1^ National Center for Magnetic Resonance in Wuhan, Key Laboratory of Magnetic Resonance in Biological Systems, State Key Laboratory of Magnetic Resonance and Atomic and Molecular Physics, Wuhan Institute of Physics and Mathematics, Innovation Academy for Precision Measurement Science and Technology, Chinese Academy of Sciences, Wuhan 430071, P. R. China.; ^2^ University of Chinese Academy of Sciences, Beijing 100049, P. R. China.; ^3^ Interdisciplinary Institute of NMR and Molecular Sciences, School of Chemistry and Chemical Engineering, The State Key Laboratory of Refractories and Metallurgy, Wuhan University of Science and Technology, Wuhan 430081, P. R. China.; ^4^ Wuhan National Laboratory for Optoelectronics, Huazhong University of Science and Technology, Wuhan 430074, P. R. China.

## Abstract

Fast collective motions are widely present in biomolecules, but their functional relevance remains unclear. Herein, we reveal that fast collective motions of backbone are critical to the water transfer of aquaporin Z (AqpZ) by using solid-state nuclear magnetic resonance (ssNMR) spectroscopy and molecular dynamics (MD) simulations. A total of 212 residue site–specific dipolar order parameters and 158 ^15^N spin relaxation rates of the backbone are measured by combining the ^13^C- and ^1^H-detected multidimensional ssNMR spectra. Analysis of these experimental data by theoretic models suggests that the small-amplitude (~10°) collective motions of the transmembrane α helices on the nanosecond-to-microsecond timescales are dominant for the dynamics of AqpZ. The MD simulations demonstrate that these collective motions are critical to the water transfer efficiency of AqpZ by facilitating the opening of the channel and accelerating the water-residue hydrogen bonds renewing in the selectivity filter region.

## Teaser

Fast timescale collective motions of transmembrane helices of AqpZ directly correlate to its water transfer efficiency.

## INTRODUCTION

Proteins are dynamic entities that exhibit motions across a broad range of timescales from femtosecond (fs) to second (s). These motions encompass both fast and slow dynamics (*1*). Fast motions include atomic vibrations, localized rotational and translational diffusive motions of small atom groups, and small-amplitude collective motions of secondary structure elements. These fast motions occur on the fs-to-microsecond (μs) timescales and contribute to rapid conformational fluctuations within specific states, leading to the generation of structure ensembles. In contrast, proteins undergo slow motions involving conformational exchanges, rearrangements, and domain diffusions that entail large conformational changes on the μs or longer timescales. These slow motions drive transitions between different states in the protein.

Functional roles of conformational dynamics on the long timescales have been well characterized by using experimental technologies, such as x-ray diffraction (*2*), nuclear magnetic resonance (NMR) spectroscopy (*3*), fluorescence resonance energy transfer (*4*), and hydrogen-deuterium exchange (*5*). Many studies have revealed the important roles of the slow dynamics in protein functions, including enzyme catalysis, signal transduction, and protein interactions. Among them, the catalytic ability of enzymes is believed to depend on the slow conformational exchanges between low-energy ground states and high-energy transition states (*6*, *7*), and signal transduction and protein-protein interactions depend on the slow conformational dynamics by which to adjust the plasticity of the interface between proteins (*8*, *9*).

Over the past 30 years, some studies suggested the indispensable roles of fast conformational dynamics (less than 1 ns) in protein functions. For instance, using x-ray diffraction, Rasmussen *et al.* (*10*) found that the substrate-binding ability of ribonuclease A was lost when the fast bond vibrations of individual atoms were settled at 212 K, whereas the ligand binding was observed when the fast dynamics was recovered at 228 K. A similar phenomenon was observed in myoglobin by x-ray crystallography (*11*). By using the time-resolved serial fs crystallography and quantum mechanics/molecular mechanics simulations, Schlichting and co-workers found that the fast motions on the fs-to-picosecond (ps) timescales couple with lower-frequency and large-scale collective motions, such as the displacements of helical segments (*12*). Henzler-Wildman *et al.* (*13*) also revealed that ps-to-ns timescale atomic fluctuations facilitate the large-scale and slower lid motions that produce a catalytically competent state by combining NMR spectroscopy and molecular dynamics (MD) simulations. The neutron scattering techniques have also been used to study the dynamic coupling between protein and solvent motions on the ps-to-ns timescales and then revealed the significance of this coupling to the protein function (*14*).

The fast collective conformational dynamics on the ns-to-μs timescales, which corresponds to the local small-amplitude collective motions of secondary structure elements, are more generally present in proteins than the slow conformational dynamics. However, the direct correlation of fast collective conformational dynamics to the protein functions remains unclear due to the great challenges in quantifying the fast collective motions of structure elements at atomic resolution and coupling fast collective motions to protein functions.

Solid-state NMR (ssNMR) spectroscopy has emerged as a powerful technique to characterize the dynamics of proteins at atomic resolution. In particular, ssNMR has distinct advantages in quantifying the timescales and amplitudes of motions on a wide range of timescales by combining spin relaxation measurement and theoretical model analysis (*15*–*22*). In particular, ssNMR can be used to characterize the conformational dynamics of all residues of proteins simultaneously, thus enabling it to characterize the fast collective motions of structure elements at atomic resolution. In addition, MD simulations can monitor the dynamics of proteins at conditions inaccessible to experiments.

In this study, we used ssNMR combining MD simulations to reveal the critical functional roles of fast collective motion in water transfer channel aquaporin Z (AqpZ), whose water translocation occurs on short timescales with a rate of about ~10^8^ s^−1^ (*23*, *24*), making it a good model protein to study the direct correlations between fast dynamics on the ns-to-μs timescales and its functions. The x-ray structures of AqpZ are available elsewhere (*25*, *26*). AqpZ functions as a homo-tetramer on membranes. Each monomeric subunit of the tetramer contains a long and narrow water channel, which is enclosed by six long and two short transmembrane α helices. AqpZ has two highly conserved functional motifs in the channel: the dual asparagine-proline-alanine motif and the aromatic and arginine selectivity filter (SF) region (*23*, *26*, *27*). The SF region constitutes the narrowest part of the water channel involving the selectivity of small molecules. Although high-resolution three-dimensional (3D) structures of AqpZ are available, the coupling between conformational dynamics and water translocation functions remains elusive. Here, we measured a large number of site-specific ^1^Hα-^13^Cα (S_CαHα_), ^15^N-^13^Cα (S_NCα_) one-bond dipolar order parameters, site-specific ^15^N rotating frame spin-lattice relaxation rate (^15^N-R_1ρ_), and ^15^N spin-lattice relaxation rate (^15^N-R_1_) by 2D and 3D ssNMR spectra covering residues of all structural elements of AqpZ. The analysis of these dynamics data by the 3D Gaussian axial fluctuation (3D GAF) model with a single timescale of motion (*18*, *28*, *29*) demonstrates that transmembrane α helices perform small-amplitude (~10°) anisotropic collective motions (ACMs) on the timescale of tens of ns. The MD simulations demonstrated that the small-amplitude collective motions of transmembrane helices are critical to water translocation across the channel, which facilitates the opening of the SF region and the formation of the hydrogen bonds (HBs) between water and key residues H174 and R189 in the SF region. The interactions between key residues H174, R189, and water molecules were further proved by the water-edited experiment. The observation of the direct correlation of fast small-amplitude collective motions on the ns-to-μs timescales to the protein function provides notable insights into the understanding of fast dynamics in protein functions.

## RESULTS

### ssNMR chemical shift assignments of backbone ^1^H_N_ of AqpZ in proteoliposomes

In a previous study, we demonstrated that AqpZ reconstituted in 1-palmitoyl-2-oleoyl-*sn*-glycero-3-phosphocholine (POPC)/1-palmitoyl-2-oleoyl-*sn*-glycero-3-[phospho-*rac*-(1-glycerol)] (POPG) proteoliposomes is functional and conformationally homogeneous (*30*). We have assigned chemical shifts of 220 out of its 231 residues by a set of ^13^C-detected 3D spectra of uniformly ^13^C,^15^N-labeled AqpZ. To further improve the spectral resolution of the ^13^C dimension and make more peaks resolved in the 2D nitrogen-alpha carbon corrected (NCA) spectra for the dipolar order parameter measurements, we prepared a ^13^C sparsely labeled AqpZ sample using 2-^13^C-glycerol as the sole carbon source in the expression of AqpZ ([^15^N, 2-^13^C-glycerol]-labeled AqpZ, 2-AqpZ). The sample preparations are highly reproducible, and chemical shifts in 2-AqpZ are almost the same as those in uniformly ^13^C,^15^N-labeled AqpZ samples.

**Fig. 1.**
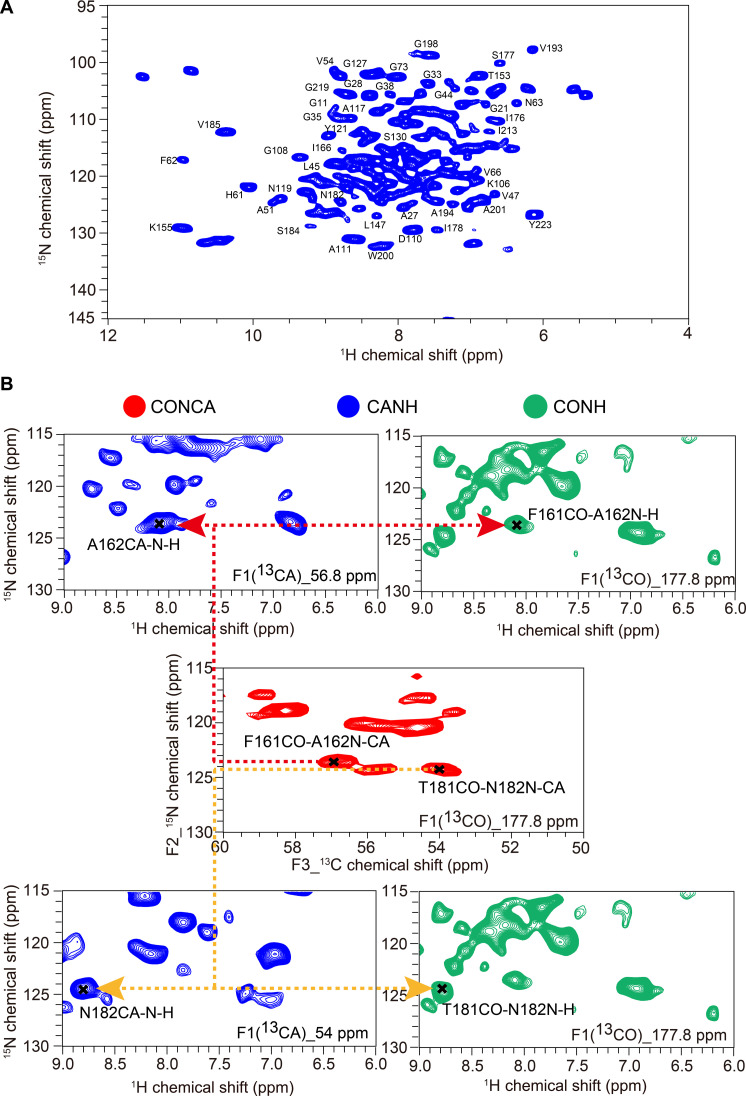
Chemical shift assignments of backbone ^1^H_N_ of AqpZ in proteoliposomes. (**A**) ^15^N-^1^H_N_ HSQC spectrum of [10%^1^H,^13^C,^15^N]-AqpZ at a MAS rate of 40 kHz by ^1^H detection. (**B**) Representative ^1^H_N_ chemical shift assignments of residues A162 and N182 by combining 3D hCANH (blue) and hCONH (green) spectra of [10%^1^H,^13^C,^15^N]-AqpZ, and 3D CONCA (red) spectra of uniformly ^13^C,^15^N-labeled AqpZ. All assigned chemical shifts are listed in table S1.

Next, for the following R_1_ and R_1ρ_ measurements, we assigned ^1^H_N_ chemical shifts by ^1^H-detected experiments using a 10% protonated and uniformly ^13^C,^15^N-labeled AqpZ sample ([10%^1^H,^13^-C,^15^N]-AqpZ). The 10% protonated (*31*) protein was expressed in the medium containing 90% D_2_O–10% H_2_O, purified and reconstituted in the buffer of 100% H_2_O and back-exchanged in the buffer containing 90% D_2_O–10% H_2_O (see Materials and Methods). Under these sample preparation conditions, all protons in the protein, including water-inaccessible protons within transmembrane domains, are diluted to a high extent. The level of 10% protonation was chosen as a compromise in terms of spectral resolution and sensitivity of ^15^N-^1^H_N_ heteronuclear single-quantum correlation (HSQC) spectra under a magic angle spinning (MAS) rate of 40 kHz by comparing the spectra of AqpZ with different protonation levels. The average linewidth of ~100 Hz [0.12 parts per million (ppm) in an 800-MHz NMR spectrometer] of ^1^H_N_ was observed in the ^15^N-^1^H_N_ HSQC spectrum of [10%^1^H,^13^C,^15^N]-AqpZ (Fig. 1A). Chemical shifts of ^1^H_N_ of 166 residues were assigned by using ^1^H-detected 3D hCANH (alpha Carbon-Nitrogen-Hydrogen corrected), hCONH (acyl Carbon-Nitrogen-Hydrogen corrected) spectra of [10%^1^H,^13^C,^15^N]-AqpZ and ^13^C-detected 3D acyl carbon-nitrogen-alpha carbon corrected (CONCA) spectra of uniformly ^13^C,^15^N-labeled AqpZ (Fig. 1B). The chemical shifts of ^15^N and ^13^C assigned in a previous study (*32*) were used in the assignments of ^1^H_N_ since the chemical shifts of ^15^N and ^13^C are well consistent with the spectra of two samples (Fig. 1B and table S1).

### The dipolar coupling–based order parameters revealed the rigidity of AqpZ in proteoliposomes

**Fig. 2.**
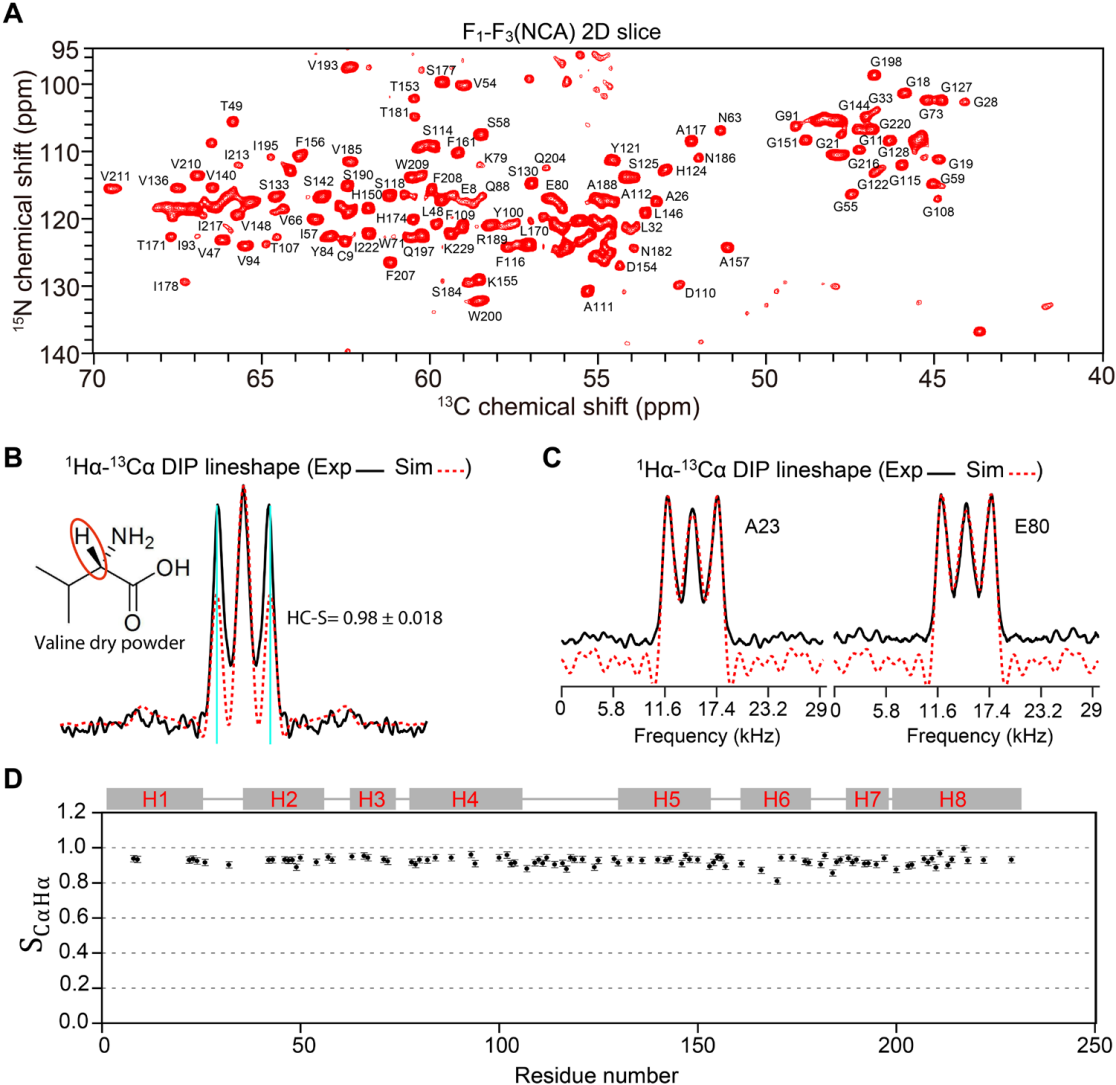
The ^1^Hα-^13^Cα dipolar order parameters (S_CαHα_) measured by 3D DIPSHIFT spectra. (**A**) The first F_1_-F_3_ 2D slice (NCA) of the 3D DIPSHIFT spectrum. Pulse sequences of 3D DIPSHIFT experiments can be found in fig. S1. (**B**) The reliability of the 3D DIPSHIFT experimental method based on the $R18_{1}^{7}$ module was evaluated by valine dry power. The experimental ^1^Hα-^13^Cα dipolar lineshapes and the corresponding best fittings are shown by black solid lines and red dashed lines, respectively. (**C**) Representative ^1^Hα-^13^Cα dipolar lineshapes extracted from 3D DIPSHIFT spectra for measuring ^1^Hα-^13^Cα dipolar couplings. The experimental dipolar lineshapes and the corresponding best fittings are shown by black solid lines and red dashed lines, respectively. All dipolar lineshapes can be found in fig. S2. (**D**) One-bond S_CαHα_ as a function of residue numbers. The secondary structure of lipid-reconstituted AqpZ is shown on top, and “H” represents the α helices. The uncertainties of S_CαHα_ were estimated by weighing the effect of dipole coupling gradient on dipole lineshapes. All one-bond dipolar order parameters are listed in table S2.

The chemical shift assignments of most residues allow us to study the residue site–specific dynamics of AqpZ. We measured ^1^Hα-^13^Cα dipolar coupling–based order parameters by 3D dipolar chemical shift correlation (DIPSHIFT) spectra of the backbone of 2-AqpZ. The pulse sequences for 3D DIPSHIFT experiments are shown in fig. S1. We first examined the reliability of 3D DIPSHIFT experimental methods by using a solid valine dry powder sample, which is rigid-limited. As shown in Fig. 2B, an S_CαHα_ of 0.98 ± 0.018 of solid valine dry powder was observed, which closely matches the theoretical expectation. In addition, our 1H-13C R1817 DIPSHIFT experiments demonstrated robustness under radio frequency (RF) inhomogeneity and resonance offset within the chemical shift anisotropy (CSA) range of protons (^C^H) (fig. S1B). Many resolved peaks are present in the first NCA 2D plane of 3D DIPSHIFT spectra owing to the high-resolution and good dispersion of NCA spectra of the 2-AqpZ sample (Fig. 2A). ^1^Hα-^13^Cα dipolar couplings were obtained by fitting experimental lineshapes (see Materials and Methods). ^1^Hα-^13^Cα dipolar couplings of 97 residues were extracted from 3D DIPSHIFT spectra (fig. S2), among which the representative two lineshapes and their best fittings are shown in Fig. 2C. Furthermore, ^15^N-^13^Cα dipolar couplings of 115 residues were extracted by a 3D z-filtered transferred-echo double-resonance (z-filtered TEDOR) experiment (see Materials and Methods), and the corresponding ^15^N-^13^Cα z-filtered TEDOR buildup curves and their best fittings are shown in fig. S3.

All measured S_CαHα_s and S_NCα_s are demonstrated in Fig. 2D, fig. S3, and table S2. S_CαHα_s and S_NCα_s are mainly distributed from 0.8 to 1.0. The residues in α helices and loops exhibit large S_CαHα_s, indicating a rigidity of AqpZ. Additionally, the large S_NCα_ values suggest that the local motions of peptide planes on submillisecond timescales are not notable.

### ^15^N-R_1_ and ^15^N-R_1ρ_s showed that ns-to-μs motions are dominant in AqpZ in proteoliposomes

Spin relaxation is highly sensitive to the amplitude and timescales of molecular motions. The R_1_ relaxation rate is sensitive to fast local motions occurring on ps-to-μs timescales, whereas the R_1ρ_ relaxation rate is sensitive to motions on the ns-to-millisecond (ms) timescales. It should be noted that both ^15^N-R_1_ and ^15^N-R_1ρ_ are influenced by ^1^H-^1^H dipolar couplings. To ensure that the observed relaxation rates are solely modulated by protein dynamics, complete suppression of ^1^H-^1^H dipolar coupling is necessary. In this study, the suppression of ^1^H-^1^H dipolar couplings was achieved by a combination of ~90% ^1^H dilution (10% protonation) of all protons in AqpZ and a fast MAS rate of 40 kHz. A MAS with a rate of above 20 kHz can be used to effectively minimize ^15^N-R_1_ rate-averaging effects from ^15^N-^15^N proton-driven spin diffusion (*33*). Furthermore, as shown in fig. S4, at a MAS rate of 40 kHz, the combination of 90% ^1^H dilution and a spinlock power of 10 kHz can efficiently suppress coherent contributions, resulting in a sufficiently small ^15^N-R_1ρ_. Therefore, the ^15^N-R_1_s and ^15^N-R_1ρ_s measured at these conditions only reflect the dynamics of proteins without interference from other factors.

**Fig. 3.**
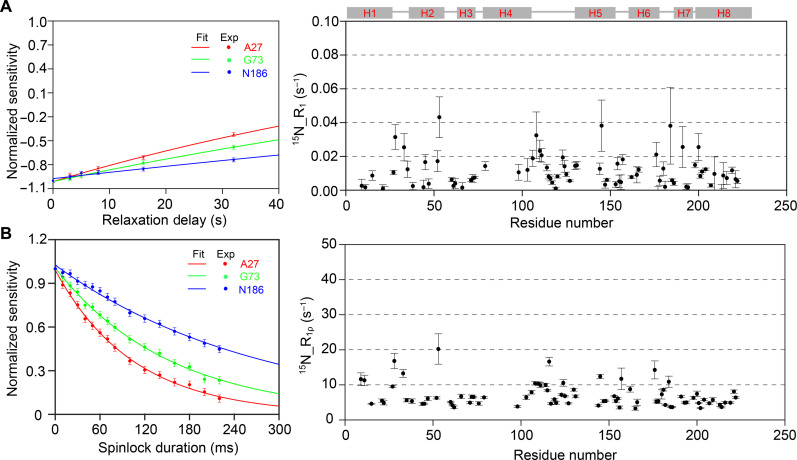
The ^15^N-R_1_ and ^15^N-R_1ρ_ measurements using a series of 2D ^1^H-detected HSQC spectra of [10%^1^H,^13^C,^15^N]-AqpZ in proteoliposomes. (**A**) Representative ^15^N-R_1_ trajectories (solid dots) and the best fittings (solid lines) of A27, G73, and N186 and site-specific ^15^N-R_1_s as a function of residue numbers. (**B**) Representative ^15^N-R_1ρ_ trajectories (solid dots) and the best fittings (solid lines) of A27, G73, and N186 and site-specific ^15^N-R_1ρ_s as a function of residue numbers. Error bars define a 95% confidence level interval. Pulse sequences for ^15^N-R_1_ and ^15^N-R_1ρ_ measurement experiments can be found in fig. S5. All ^15^N-R_1ρ_ and ^15^N-R_1_ trajectories and the best fittings can be found in figs. S6 and S7. All ^15^N-R_1ρ_s and ^15^N-R_1_s are listed in table S2.

To measure site-specific ^15^N-R_1_ and ^15^N-R_1ρ_ values, we used pulse sequences as depicted in fig. S5. We acquired a series of 2D ^15^N-^1^H_N_ HSQC spectra with varying inversion recovery durations or spinlock power durations in the ^15^N channel, as described in Materials and Methods. The high-resolution and well-dispersed 2D HSQC spectra of [10%^1^H,^13^C,^15^N]-AqpZ allowed us to extract ^15^N-R_1_ and ^15^N-R_1ρ_ values for 79 residues distributed across all secondary structure elements of AqpZ (figs. S6 and S7 and table S2). Figure 3A illustrates the representative ^15^N-R_1_ trajectories and their corresponding best fits for residues A27, G73, and N186. Additionally, the site-specific ^15^N-R_1_ values are plotted as a function of residue numbers. For residues located in α helices, the ^15^N-R_1_ values range from 0 to 0.05 s^−1^ with low uncertainties, indicating that local motions on subnanosecond–to–20-ns timescales are not notable in AqpZ (fig. S8). In Fig. 3B, the representative ^15^N-R_1ρ_ trajectories and their best fits for residues A27, G73, and N186 are shown, along with ^15^N-R_1ρ_ values as a function of residue numbers. Most residues in α helices exhibit ^15^N-R_1ρ_ values ranging from 3 to 8 s^−1^ with low uncertainties. However, a few residues in loops or at the termini of helices have ^15^N-R_1ρ_ values distributed from 8 to 30 s^−1^ with relatively higher uncertainties. These observations suggest that the timescales of motions within AqpZ span from 20 to 110 ns or from 100 μs to 1 ms (fig. S8).

We further used the simple model free (SMF) model to analyze the motion timescale of each residue within AqpZ. Most residues, except some residues in loops or in helix terminals, exhibit χ^*2*^ values below 10, showing the reliability of the analysis (fig. S9). The results revealed that the dominant motion timescale of most residues within the helices is distributed between 30 and 90 ns, which is consistent with narrow distributions of ^15^N-R_1_ values ranging from 0 to 0.05 s^−1^ and ^15^N-R_1ρ_ values ranging from 3 to 8 s^−1^ (fig. S8). The similarity in motion timescale of different residues probably reflects the correlation or collective motion characteristics between residues. This conjecture can also be supported by ssNMR spectra. For example, the high resolution observed in the ssNMR spectra of AqpZ suggests that local motions on the μs-to-ms timescales are not prominent; otherwise, these motions, especially conformational exchanges, can deteriorate spectral resolution. Furthermore, the observation of large S_NCα_s (0.8 to 1.0) suggests that local motions of the peptide plane on submillisecond timescales are not notable in AqpZ (fig. S3). To investigate the dominant modes of motion within AqpZ, we conducted MD simulations.

### MD simulations revealed the presence of collective motions within AqpZ

**Fig. 4.**
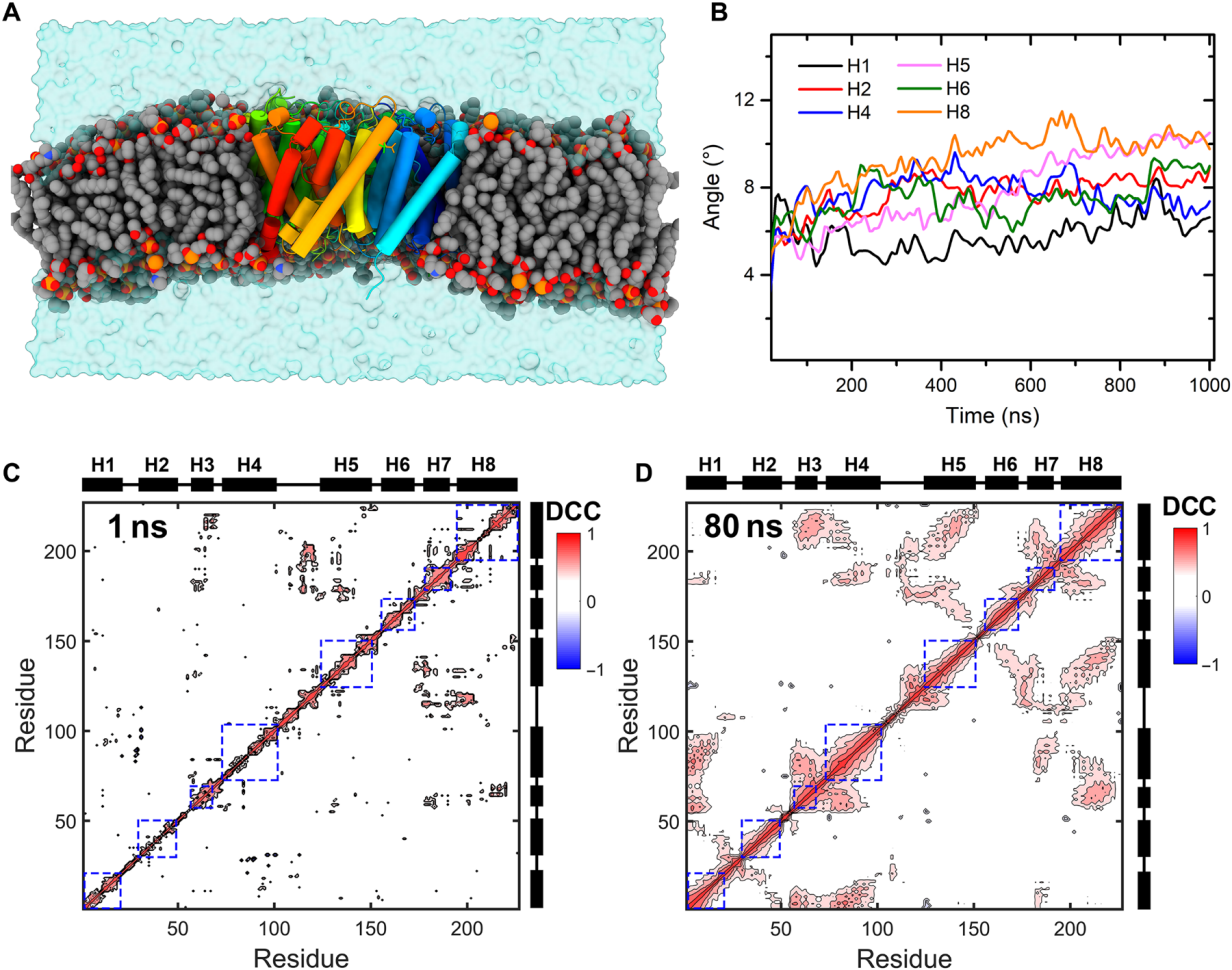
MD simulations revealed the collective motions of transmembrane helices in AqpZ proteoliposomes. (**A**) The all-atom model of AqpZ membrane in the simulations. The helical structures of AqpZ are shown in cylinders, the POPC/POPG lipids are represented by balls, and the phosphorus atoms are colored orange. The lipid bilayer and protein are hydrated in the water box, which is shown in the blue translucence glass. (**B**) The fluctuation of helices’ orientation. The angles are defined as the helices rotating around their initial directions. The small-amplitude rocking of transmembrane helices is also shown in fig. S10, and the angle fluctuations in a 1-ns time interval of all the helices are also shown in figs. S11 to S16. The DCC map of AqpZ derived from the 1-ns MD simulation trajectory (**C**) and the 80-ns MD simulation trajectory (**D**). The DCC values reveal the correlation between residues within the same transmembrane helices. The same-direction motions with positive DCC values are colored red, and the opposite direction motions between the residue pairs are colored blue. The positions of helices are given beside the axis, the helices are presented by the black bars, and the loops that connect them are presented by black lines.

In MD simulations, the initial structures of AqpZ are built based on the high-resolution ssNMR structure of AqpZ reconstituted in the synthetic membrane bilayers (*30*). The proteins are inserted into the membrane bilayers composed of POPC and POPG (in the ratio of 3:1, w/w), which is consistent with the ssNMR experiments (Fig. 4A). The μs-long MD simulations revealed the existence of small-amplitude motions of the transmembrane helices of AqpZ within the lipid bilayers (Fig. 4B and fig. S10). The dynamic cross-correlation (DCC) based on the MD simulation revealed the presence of collective motions within the transmembrane helices of AqpZ. The DCC values give the correlated motions of residue pairs by calculating the covariance between the fluctuations of these residues (*34*, *35*). In the short time intervals (i.e., 1 ns), no correlated motions were present within the transmembrane helices because the DCC values are close to zero for the non-adjacent residues (Fig. 4C). In contrast, DCC values between lots of separated residues in the transmembrane helices are remarkable in the time interval of 80 ns (Fig. 4D), which means a higher degree of correlations present in the motions between residues within the helices on such timescales. The notable correlative motions of residues in the transmembrane helices indicate the collective motions of residues in these structure elements. In the simulations, the integrality of transmembrane helices is maintained, but the orientations of the helices fluctuate in angles less than 10° related to the initial structures. The angle fluctuations in the 1-ns time interval of all the helices are also smaller than 10° (figs. S11 to S16), which means the rotations of the helices are small-angle rocking without large conformational changes.

### Analysis of order parameters, ^15^N-R_1_, and ^15^N-R_1ρ_s using the 3D GAF model further revealed the presence of collective motions within AqpZ

**Fig. 5.**
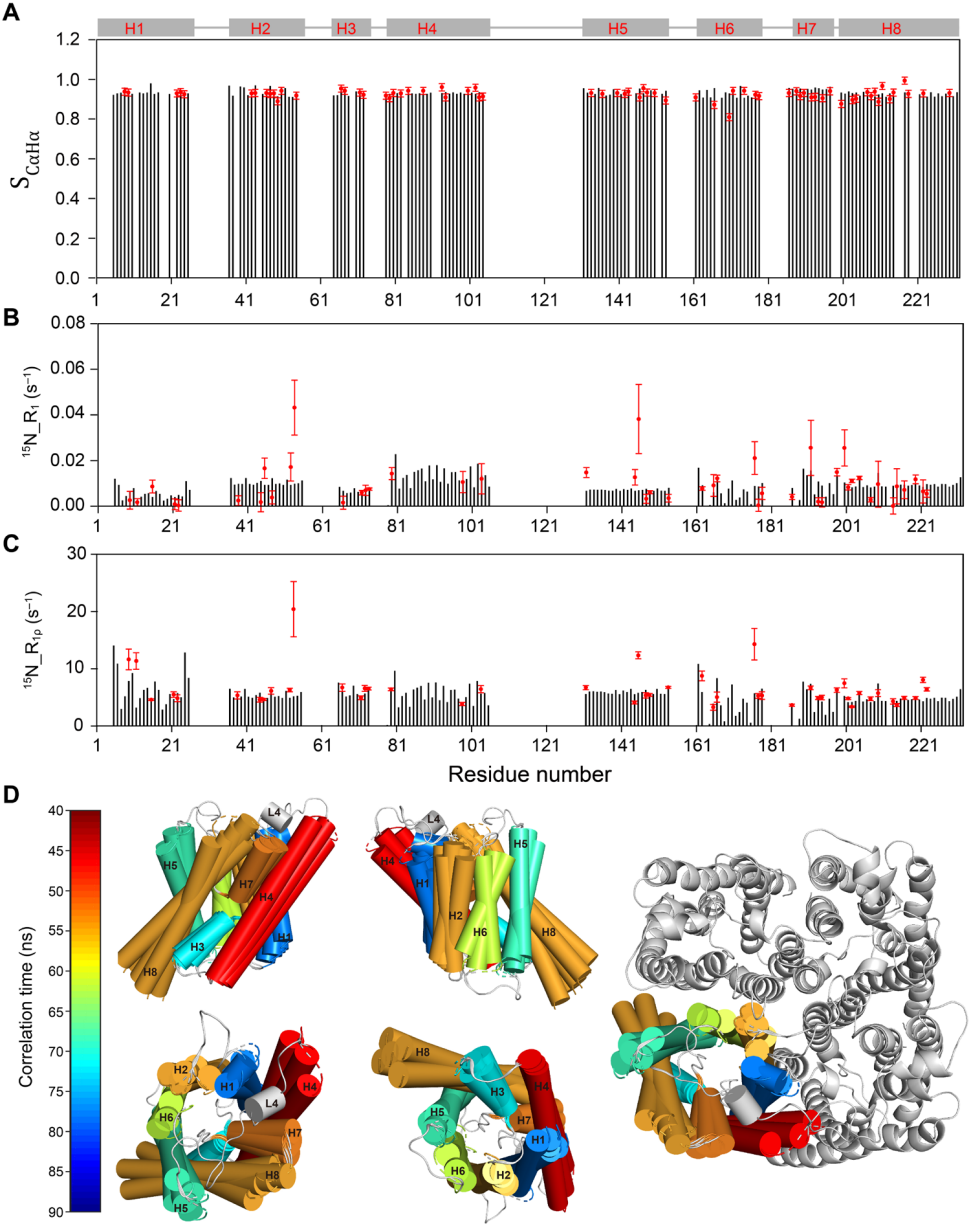
The motional details of ACMs were obtained by analyzing S_CαHα_s, ^15^N-R_1ρ_s, and ^15^N-R_1_s using the 3D GAF model. (**A** to **C**) The comparisons of site-specific dynamic parameters (S_CαHα_, ^15^N-R_1ρ_, and ^15^N-R_1_) between experimental results (red dots) and theoretical results calculated by the 3D GAF model (gray bars). (**D**) Motional amplitudes, directions, and timescales of different secondary structural segments of AqpZ obtained from fittings of S_CαHα_, ^15^N-R_1ρ_, and ^15^N-R_1_. The amplitudes and directions of ACMs shown in the figure are indicated with conformations corresponding to rotation extremes from the average position. The effective motional timescales are indicated in different colors. All the best-fitted dynamic parameters for the ACMs of AqpZ and the evaluation of 3D GAF calculation quality are listed in table S3.

To further investigate whether the MD results are supported by experimental data, we analyzed these data by the 3D GAF model with a single effective timescale of motion (*18*, *28*). The prerequisite for the 3D GAF model is the presence of ACMs within the helices or motions with long-range correlations. If there is no long-range correlated motion within the protein, then it is not possible to obtain good fittings with the 3D GAF model. We used the ssNMR structural model of AqpZ in the analysis (*30*). Since the timescales of motions are distributed in the ns-to-μs range (fig. S9), which is the range sensitive to both ^15^N-R_1_ and ^15^N-R_1ρ_ (fig. S8), we included three experimental parameters (S_CαHα_, ^15^N-R_1_, and ^15^N-R_1ρ_) in the calculations. The details of the fittings using the 3D GAF model are provided in Materials and Methods, and the fitted results are shown in Fig. 5 and table S3.

We compared the experimentally measured S_CαHα_, ^15^N-R_1_, and ^15^N-R_1ρ_ with the corresponding theoretically calculated ones based on the 3D GAF model. As shown in Fig. 5 (A to C), S_CαHα_, ^15^N-R_1_, and ^15^N-R_1ρ_ of most residues in α helices are well consistent between experimental data (red dots) and the fitted ones (gray bars), indicating that the transmembrane helices exhibit notable ACMs. The quality of the 3D GAF fitting was evaluated with the reduced χ^2^ (χ_red_^2^). In table S3, all transmembrane helices have χ_*red*_^*2*^ values of less than 6.0, suggesting that the 3D GAF model is a good model for interpreting our data. In the 3D GAF model, only the collective motions are considered. If ^15^N-R_1_ and ^15^N-R_1ρ_ are influenced by additional local slow motions that dominate the stochastic fluctuations, the experimental data cannot be well fitted. Therefore, the deviations between experimental results and fitted ones of a few residues probably indicated the presence of additional internal local motions in addition to collective motions.

The timescales, amplitudes, and directions of ACMs analyzed by the 3D GAF model are depicted in Fig. 5D, and all the fitted values are listed in table S3. Different α helices of AqpZ perform a small-amplitude (~10°) anisotropic rocking, which is consistent with the results of the MD simulation (Fig. 4). The ACMs of AqpZ are also similar to those in studies of Anabaena sensory rhodopsin (ASR) (*18*) and *Klebsiella pneumoniae* (KpOmpA) (*19*). Although the amplitudes of the ACMs are likely overestimated as we explicitly neglect fast ps-ns motions that also contribute to the measured S_CαHα_, local fast ps-ns motions do not dominate because of such small ^15^N-R_1_ values. In addition, no ^15^N-R_1ρ_ relaxation will occur if local fast ps-ns motions dominate. Therefore, the measured S_CαHα_s are mainly modulated by ns-to-μs small-amplitude (~10°) anisotropic rocking of α helices. Generally, the timescales of the ACMs within the α helices of AqpZ are distributed from 40 to 90 ns, which is consistent with the results of SMF calculations (fig. S9) and DCC values in the MD simulation (Fig. 4).

It is difficult to extract a specific type of motion solely from ssNMR dynamic parameters since the correlations between dynamics data and internal motions of proteins are not straight. In this study, a combination of the analysis of dynamics data by the 3D GAF model and MD simulation results has revealed the presence of ACMs within AqpZ, but this does not mean that there are no local motions within AqpZ. However, the high DCC values in the MD simulations and the good 3D GAF fitting indicate that the local motions within AqpZ are not notable.

### MD simulations revealed that the collective motions of transmembrane helices are critical to water permeability

**Fig. 6.**
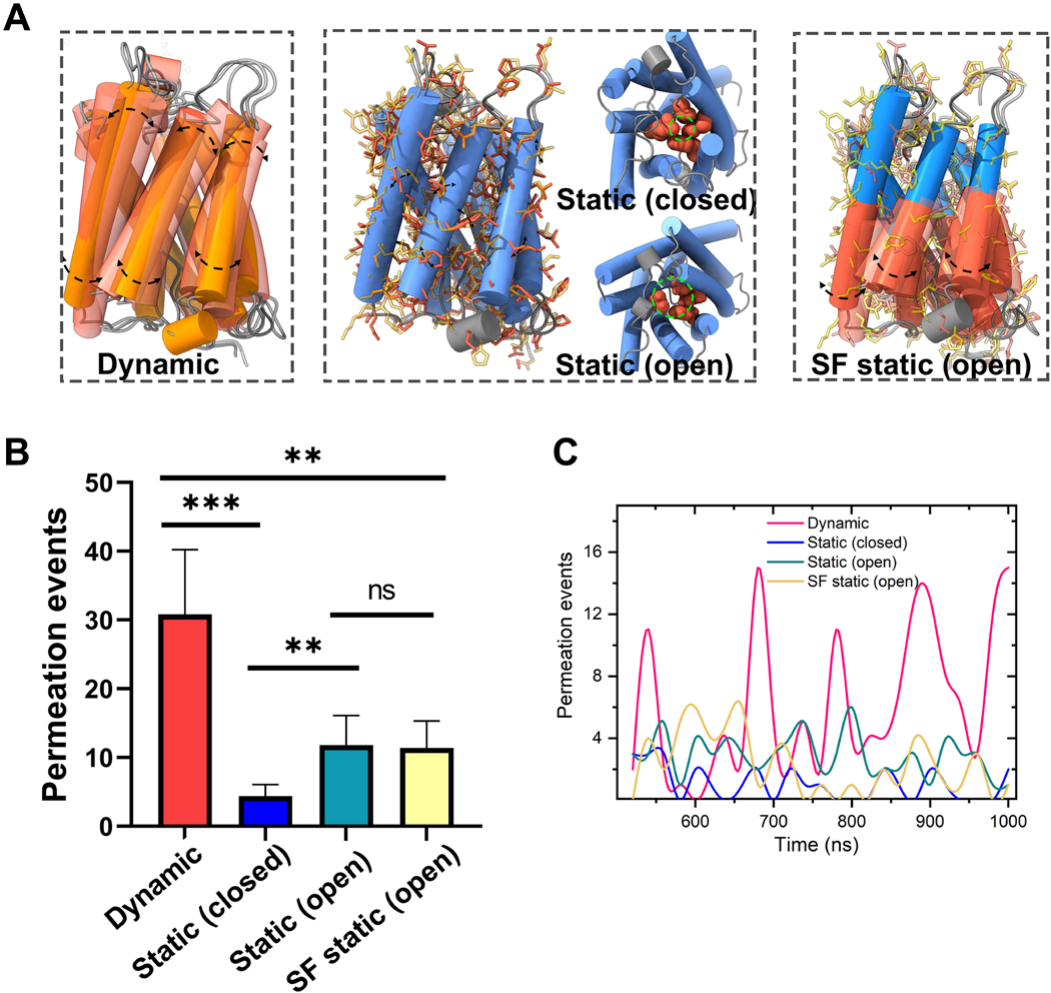
MD simulations revealed that the collective motions of transmembrane helices dominate water permeability. (**A**) The different dynamic structural models of AqpZ. In the “Dynamic” model, all the atoms of AqpZ move freely. In the “Static (closed)” models, the water translocation gate near the SF region is closed in the initial structure of simulations, and the position restraints were added on the backbone atoms of the protein. Instead, the SF gate is open in the “Static (open)” model. In the “SF static (open)” model, the position restraints are only added on the backbone atoms of the SF region, and the initial structure of the SF region is open. (**B**) The average water permeation events per 100 ns. The water permeation event is defined as the complete crossing of the water molecule from one side of the lipid bilayer to the other side. The error bars are the deviation of the permeation events in the last 500-ns simulations. ***P < 0.001, **P < 0.01; ns, not significant. (**C**) The water permeation events as a function of simulation time; the permeated water events that happened in every 20 ns of the simulations were accounted for.

To investigate the roles of the small-amplitude rocking of transmembrane helices to the water permeation, we designed four kinds of systems (Fig. 6A): All atoms can freely move in the dynamic system; position restraints were added on the backbone atoms to fix the backbone motions, and the initial structure of the SF region is closed [hole radius smaller than 1.4 Å, which is the typical sphere radii of the water molecules (*26*, *36*)] in the static (closed) system; position restraints were added on the backbone atoms, and the initial structure of the SF region is open in the static (open) system; and position restraints were only added on the backbone atoms of the SF region, and the initial structure of the SF region of is open in the SF static (open) system.

The amount of permeated water molecules passing the water channel within AqpZ in different systems is given in Fig. 6B. The freely dynamic AqpZ has the highest water translocation efficiency. On average, about 30 water molecules were transferred from one side of the membrane to the other side by AqpZ every 100 ns. For comparison, only about four water molecules were transferred by the restrained AqpZ with a closed initial structure. Even though the initial structure of the SF region is open, the water permeation is also relatively low when the motions of the backbone are restrained. The comparison of water translocation efficiencies in these four different dynamics systems indicates that the small-amplitude rocking of transmembrane helices is crucial to water transfer. The profile of water permeation number by AqpZ as a function of time is periodically oscillating (Fig. 6C). The interval time between peaks is about 100 ns and close to the frequency of the protein collective motions revealed by ssNMR, which indicates that the small-amplitude rocking of transmembrane helices on a timescale ranging from 40 to 90 ns may be the rate-limiting step of water transfer.

### The molecular mechanism of fast dynamics in determining water translocation

**Fig. 7.**
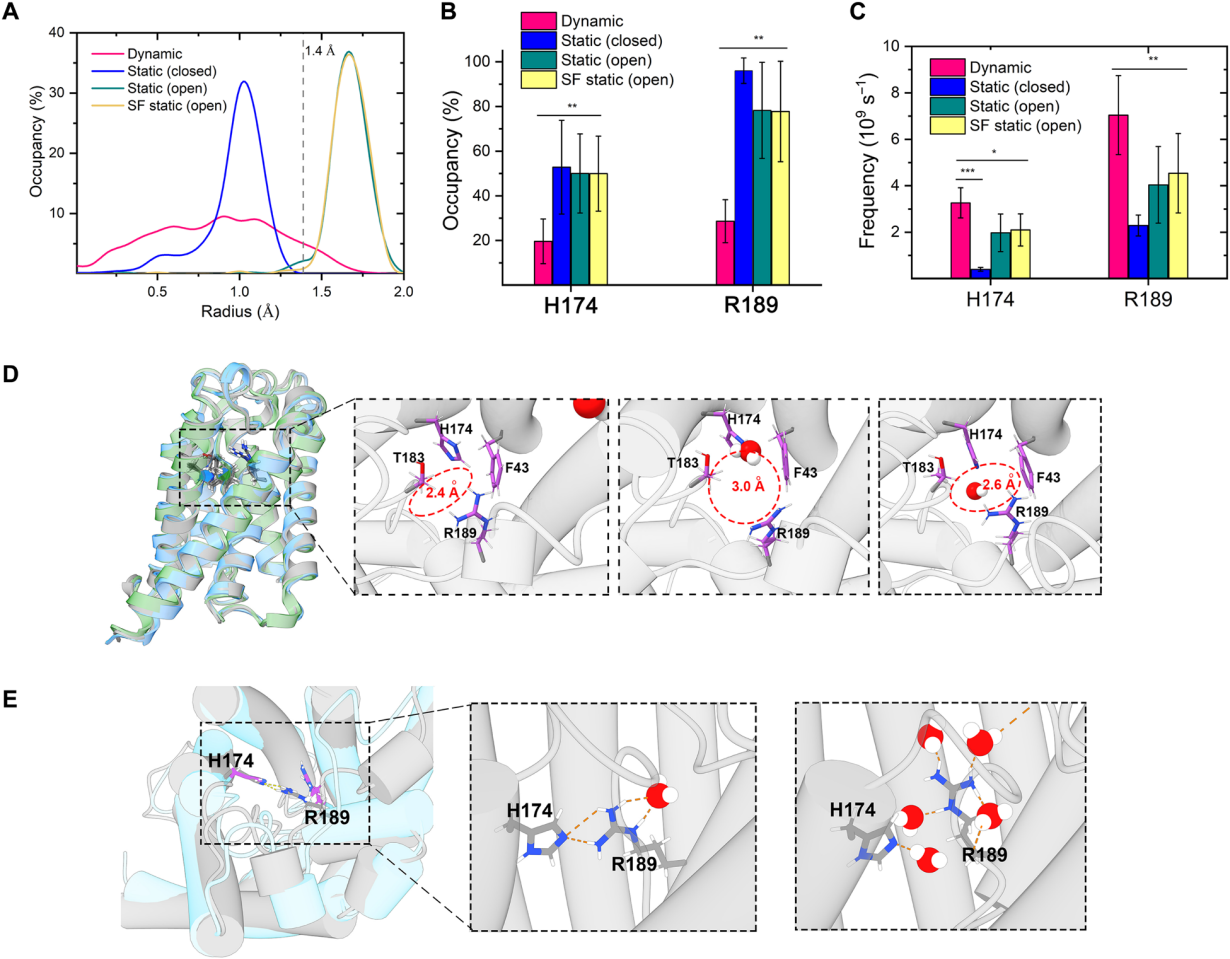
The molecular mechanism of fast dynamics facilitating water translocation. (**A**) The hole radius distribution of the SF region. The values correspond to the radius of SF regions, which are the narrowest bottleneck of the translocation channel. The dashed line represents the typical sphere radii (1.4 Å) of the water molecules. (**B**) The HBs occupancy of residues H174 and R189 formed with other residues of AqpZ. The errors are calculated over the last 500-ns simulations. ***P < 0.001, **P < 0.01, *P < 0.05. (**C**) The HBs formation frequency of H174 and R189 with water molecules, which accounts for how many different water molecules interact with these residues via HBs. The errors are calculated over the last 500-ns simulations. (**D**) The dynamics of the transmembrane helices regulate the channel radii around the SF region. Three representative structures are shown, which correspond to the SF-closed structure (radius equal to 1.2 Å), the SF-open structure (radius > 1.4 Å), and the semi-open structure, respectively. (**E**) The HBs between H174 and R189 would impede the approaching of water molecules and residues-solvate HBs formation (left). On the other side, the breaking of H174-R189 HBs facilitates the interaction between the residues and water molecules (right). Helices are rendered as cartoon models, SF residues are rendered as stick models, waters are rendered as sphere models, and red dotted lines represent the HBs.

In the x-ray structures, if the radius of the pore constriction (~1.0 Å) of the SF region is narrower than the size of water (van der Waals radius ~ 1.4 Å) (*26*, *36*), then how do the conformational dynamics of AqpZ facilitate the water molecule to pass through such a narrow pore selectively and efficiently? To investigate the molecular mechanism of conformational dynamics in determining the water-translocation functions of AqpZ, we first analyzed the relations between the hole size of the channel and the water-transfer rate by MD simulations. We found that the hole size of the SF region can be regulated by the ACMs of α helices. As shown in Fig. 7 (A and D), the freely dynamic AqpZ has the widest distribution of hole size, and about 15% of conformations in the SF-open state in the hole radii are larger than the size of water molecules. In contrast, the restrained systems have confined hole size distributions in which the hole radii are determined by the initial structures. During MD simulations, for the Static (closed) or Static (open) system, less than 1% of conformations are in the SF-open or SF-closed state, respectively, which means that the backbone motions of transmembrane helices greatly affect the configuration space of the side-chain residues in the SF region. Although the water translocation rate of AqpZ in Static (closed) systems is about threefold lower than that in the Static (open) system, the water translocation rate of AqpZ in Static (open) and SF static (open) systems is still about threefold lower than the fully unrestrained AqpZ (Fig. 6B), which showed that the motions of the backbone are critical to the translocation rate by regulating the SF-region size of the channel of AqpZ.

In aquaporins, water permeates across the transmembrane by a continuous water file, with rapid alternately forming and breaking HBs between water molecules and pore-line residues (*23*, *26*, *37*, *38*). To further investigate how motions facilitate water transfer, we characterized the correlation between motions and rapid alternating of HBs network in the channel of AqpZ by MD simulations. The SF region consists of four residues: F43, H174, T183, and R189, of which H174, T183, and R189 create a hydrophilic triangle opposite the hydrophobic F43. We found that the formed HBs between the important residues (H174 and R189) in the SF region and other residues in freely dynamic AqpZ are transient and unstable, whereas these HBs in the restrained AqpZ are more stable (Fig. 7, B and E). The residue-residue HB interactions limited the formation of HBs between the residues and water molecules. In the freely dynamic protein, the residue-water HBs are more efficiently formed (Fig. 7, C and E) and therefore more water molecules interact with the residues H174 and R189, which would guide the passing through the channel. The critical roles of H174 and R189 and their corresponding HBs revealed by our MD simulations are consistent with the previous experimental results. Kosinska Eriksson *et al.* (*37*) found that arginine and histidine in the SF region involve four water-binding sites in the SF region. Savage *et al.* (*36*) found that stepwise mutation of the AqpZ SF resulted in a noticeable decrease in water permeability by introducing a more hydrophobic SF.

**Fig. 8.**
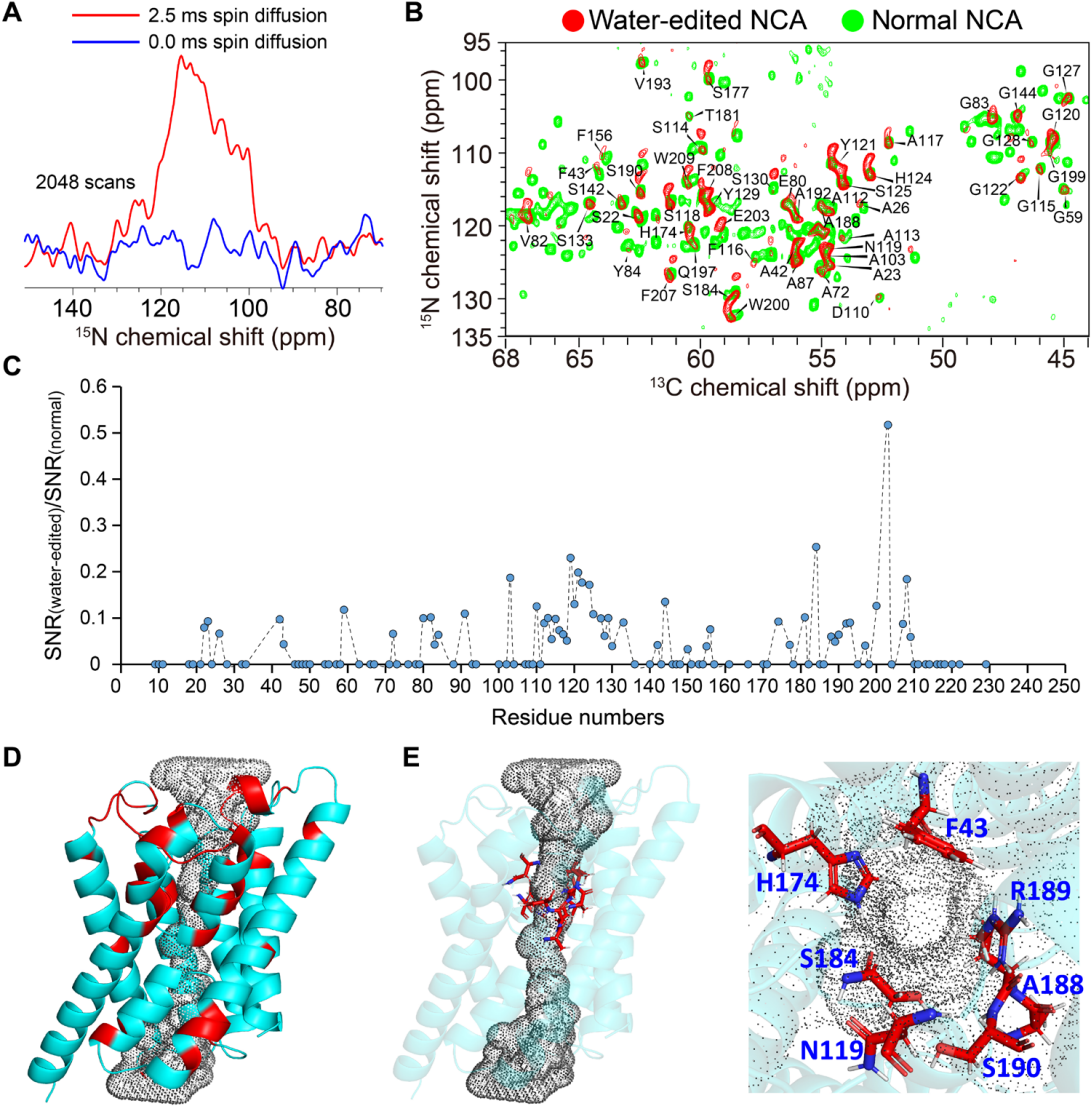
Protein-water interactions detected by the water-edited experiment. (**A**) Water-edited 1D ^15^N spectrum with ^1^H-^1^H spin diffusion times of 0 ms (blue) and 2.5 ms (red), the number of scans of both were 2048, and the line broadening factors of both were set to 100 by TopSpin. (**B**) The water-edited 2D NCA spectrum with a ^1^H-^1^H spin diffusion time of 2.5 ms (red) is superimposed on the normal 2D NCA spectrum (green). (**C**) The ratio of the SNR of the signal in the water-edited spectrum to the normal spectrum. Considering the influence of noise level on signal confirmation, we specify the SNR as zero for water-edited signals with SNR less than 5.0. (**D**) All residues with [SNR(water-edited)/SNR(normal)] larger than 3% were marked in the monomer structure of AqpZ (cyan, cartoon mode by PyMOL), and the black dots draw the shape of the water channel. (**E**) Residues (red, stick mode by PyMOL) close to the water channel and present in the water-edited 2D NCA spectrum were highlighted in the monomer structure of AqpZ.

To verify that water permeability is dependent on protein-water interactions regulated by protein dynamics, we investigated the direct protein-water interactions. In our previous study, we demonstrated that AqpZ molecules are functional in POPC/PG proteoliposomes, indicating the presence of water molecules in the water channel of AqpZ. To examine residue site–specific protein-water interactions, we conducted a water-edited 2D NCA experiment (fig. S17) (*39*). In this experiment, the ^1^H signal of water was selected using a Gaussian-shaped selective pulse and a ^1^H T_2_ filter. The water signals were then transferred to the protein via ^1^H-^1^H spin diffusion. To ensure that the protein signals only originated from the water molecules, a short ^1^H-^1^H diffusion time of 2.5 ms and a ^1^H-^15^N CP contact time of 200 μs were used to prevent remote ^1^H spin transfer, and a T_2_ filter of 0.9524 ms (10 times the rotor period) was applied to entirely eliminate the protein’s proton signals. A control experiment with zero ^1^H-^1^H diffusion time confirmed that protein signals were completely removed by a Gaussian-shaped selective pulse and a ^1^H T_2_ filter, and only signals from water molecules remained (Fig. 8A). The water-edited 2D NCA spectrum is shown in Fig. 8B, and the ratio of the signal-to-noise ratio (SNR) of the signal in the water-edited spectrum to the normal spectrum is shown in Fig. 8C. In Fig. 8C, the SNR of most water-edited NCA signals accounts for about 3 to 25% of the SNR of normal NCA signals, which is consistent with the short ^1^H-^1^H diffusion time of 2.5 ms. In Fig. 8C, the [SNR(water-edited)/SNR(normal)] values of 51 residues are larger than 3%, indicating that water molecules are in proximity to [<3.5 Å (*40*)] these residues. In Fig. 8D, 51 residues with [SNR(water-edited)/SNR(normal)] larger than 3% were marked in the monomer structure of AqpZ. Among these residues, 44 residues that generate water-edited signals are located at the membrane interface, where these regions can directly contact water, and 7 residues are distributed in the water channel (Fig. 8D), including the SF residues (F43 and H174) (Fig. 8E). In this work, all ssNMR experiments were performed using the 2-AqpZ sample. In the 2D NCA spectrum of this sample, T183 was not present due to the sparse labeling pattern of 2-AqpZ, but we observed the presence of S184 in the water-edited 2D NCA spectrum (Fig. 8E). A188 and S190 were present in the water-edited 2D NCA spectrum (Fig. 8E), although R189 was not present. In addition, our previous work directly observed the chemical exchange between the side chain of R189 and water (*30*). These results confirm the presence of direct protein-water interactions within the water channel, including interactions between H174, R189, and water molecules.

## DISCUSSION

Motions on all different timescales, including short and long timescales, possibly contribute to protein functions. The water transporting through aquaporin is fast in the rate close to ~10^8^/s (the transporting is on the ns-to-μs timescales) (*23*, *24*). However, the SF region of aquaporin is very narrow in the native conformation, which only allows the passing of single-chain water. The high transporting efficiency requires either the slow large conformational changes to allow amounts of water molecules to pass in a very short time or the fast transitions between the open and closed structures. The large conformational exchanges on the long timescales (greater than μs) are excluded by ssNMR spectral resolution, which means that the high transition rate is determined by the fast motions of AqpZ. The experimentally observed high S_CαHα_s and S_NCα_s and small ^15^N-R_1_ indicate that internal fast motions of the backbone on the subnanosecond timescales are highly restricted. In the MD simulations, when the position restraints were added to the backbone atoms in the “Static” model to freeze the motions of the backbone, the fast motions of side chains, including the rotational and translocation diffusive motions, are unlimited. However, with these motions of side chains, water translocation was almost inefficient in the Static model, suggesting that the fast dynamics of side chains are not critical to the water translocation. In contrast, as the ACMs on the ns-to-μs timescales were observed to dominate the motions of the backbone by ssNMR experiments, the motions of the backbone to water translocation, revealed by MD simulations, are also mainly contributed from ACMs. In addition, the numbers of transferred water fluctuated with a period of 100 ns, which is consistent with the idea that the ACMs on a timescale ranging from 40 to 90 ns are the rate-limiting step of water transfer.

How do the collective motions of the backbone affect the water translocation rate in the water channel of AqpZ? In this study, we observed direct interactions between pore-line residues and water molecules using water-edited experiments. These interactions are likely regulated by the internal backbone collective motions. While these regulations could be detected by changing the temperature, large changes in temperature may pose challenges to ssNMR experiments, such as reduced detection resolution and sensitivity of membrane proteins. Alternatively, using MD simulations, we demonstrated that HBs between water and pore-line residues, which are one of the major interactions between the protein and water, are regulated by backbone collective motions. Compared with the restrained inert systems, the inner HBs between the residues at the important SF region in the fully free AqpZ are more unstable. After the breaking of HBs between H174, R189, and the other residues, the unoccupied residues H174 and R189 are more possible to form HBs with water molecules, which promotes the water file formation in the channel and favors the water molecule translocation. Thus, by combining ssNMR experiments and MD simulations, we demonstrated that the residue-water HBs in AqpZ are regulated by the internal backbone collective motions, which, in turn, affects the rate of water translocation. Our studies provide notable insights into the molecular basis of conformational dynamics to the protein functions.

The collective motion in proteins has been studied systematically by various researchers. Ladizhansky and co-workers conducted research on the membrane protein ASR and discovered that its transmembrane helices exhibit collective motion spanning ns to μs (*18*). Pintacuda and colleagues reported that the β barrel structure of KpOmpA in the phospholipid membrane displays small-amplitude collective motion on the ns timescale (*19*). Schanda and co-workers observed that ubiquitin exhibits rocking motions of varying amplitude on timescales ranging from 0.1 to 100 μs in different crystal forms (*41*). Emsley and co-workers found that, at temperatures above 220 K, anisotropic motions involving entire peptide units become dominant and functionally relevant (*42*). In this study, we revealed a direct coupling of the fast collective motions of transmembrane α helices on the ns-to-μs timescales to membrane protein functions. In previous studies, this direct correlation has not been revealed due to either the lack of the residue site–specific characterization of fast collective motions in proteins or the lack of the functional relevance of the fast collective motions of proteins. Our advance in protein dynamics is achieved by investigating a suitable model protein AqpZ using a combination of advanced ssNMR methodologies and MD simulations. First, in ssNMR experiments, advances in ssNMR methodologies and theoretical model analysis enable the identification of specific motion and the quantification of motional timescales in proteins. The high quality of the ssNMR spectra with high-resolution, high-sensitivity, and good chemical shift dispersion of AqpZ in this study allowed the analysis of the dynamics of all structural domains of the protein. Second, in MD simulations, motions in some structural domains can be flexibly restrained at different degrees, enabling one to directly correlate specific motions to protein functions. These diverse conditions in MD simulations are inaccessible to experiments. In addition, with monitoring of protein conformations in the dynamic process at atomic resolution, MD simulations showed a great advantage in elucidating molecular mechanism of the dynamics events. Third, it is important that the timescales of water translocation of AqpZ is close to that of the fast collective motions, making it possible to directly correlate a collective motion of transmembrane helices to water translocation.

Small-amplitude ACMs on the ns-to-μs timescales are thermal motions with very little activation energy, which enables these proteins to adopt special stable conformations and translocation water molecules only by osmotic pressure without costing extra energy. In addition to this direct facilitation of the fast motions to protein functions, indirect contributions of the fast motions to protein functions have been revealed in a way that is related to conformational entropy (*43*–*45*). Therefore, the fast thermal motions in proteins should not be ignored in understanding the functional mechanism in proteins. In summary, we have revealed the direct facilitation of fast collective motions on the ns-to-μs timescales to the water translocation function of AqpZ. The excellent quality of ssNMR spectra of AqpZ enabled us to characterize the dynamics of proteins at atomic resolution by measuring many residue site–specific dipolar order parameters, ^15^N-R_1_ and ^5^N-R_1ρ_. The high dipolar order parameters close to 1.0 show the high rigidity of AqpZ. Analysis of these dynamic data using the 3D GAF model demonstrated that small-amplitude (~10°) ACMs of transmembrane α helices with timescales of tens of ns are dominant to the dynamics of AqpZ. The μs-long MD simulations using different restraint conditions demonstrated that the small-amplitude (~10°) rocking of transmembrane helices lubricates water molecules to pass through the water channel by facilitating the forming of HB between water and pore-line residues and by alternately enlarging narrow constriction of the pore. The interactions between water and pore-line residues have been further proven by the water-edited experiment. Since small-amplitude ACMs on the ns-to-μs timescales are thermal motions, they enable the AqpZ to adopt elastic conformations and facilitate the translocation of water molecules only by osmotic pressure without undergoing large conformation rearrangements and costing extra energies. The dynamic personality of AqpZ and the functional role of ACMs disclosed in this study promote our understanding of the dynamic molecular mechanism of AqpZ. Our study provides a direct correlation of fast collective motions of transmembrane helices on the ns-to-μs timescales to the membrane protein functions.

## MATERIALS AND METHODS

### Sample preparations for ssNMR experiments

Three AqpZ proteoliposome samples with a lipid (POPC/POPG, 3:1, w/w)–to–protein ratio of 1.25 (w/w) were used in this study. All ^13^C-detected 3D DIPSHIFT experiments were conducted using one 2-AqpZ sample. ^1^H-detected 3D hCANH, hCONH spectra (*46*) for ^1^H_N_ resonance assignments, and the measurements of ^15^N-R_1ρ_ were carried out using one [10%^1^H,^13^C,^15^N]-AqpZ sample. The 3D CONCA spectrum was acquired using a U-^13^C,^15^N-labeled AqpZ sample. AqpZ in all samples was expressed in *E. coli* BL21 (DE3) strain by a modified “dual media” approach (*47*).

The protocols of protein expression, purification, and reconstitution were repeatable for U-^13^C,^15^N-labeled AqpZ by our laboratory (*30*). The expression vector pET H6-AqpZ encodes the full-length *E. coli* aqpz gene (GenBank ID U38664.1) with an N-terminal 6×His tag sequence of MGSSHHHHHHEF. The wt-AqpZ plasmids were expressed in *E. coli* BL21 (DE3) strain by the dual-media approach. Briefly, several expression colonies were inoculated into 100 ml of LB medium with ampicillin (100 μg/ml) for cultivation expansion overnight. Next, 10 ml of *E. coli* cell suspension was transferred into 1 liter of LB medium, and the cells were harvested by centrifugation at 6000*g* for 10 min until OD_600_ (optical density at 600 nm) reached 0.6 to 0.8. After the cells were recovered in 250 ml of M9 medium [^13^C glucose (4 g/liter) and ^15^N NH_4_Cl (1 g/liter)] by shaking at 220 rpm at 310 K for 30 min, 1 mM isopropyl β-d-1-thiogalactopyranoside was added to induce expression at 310 K for 5 hours. To purify AqpZ, the cells were lysed by ultrasonication, and the unbroken cells were removed by low-speed centrifugation at 6000*g* for 10 min. The total cell membranes were collected by high-speed centrifugation at 48,384*g* for 1 hour and then solubilized into 1% sodium *N*-lauroyl sarcosinate at 277 K overnight. The purification was carried out using nickel affinity chromatography. The yield of the expression by the dual-media approach was 36 to 44 mg of pure AqpZ per liter of M9 medium culture.

The protocols of protein expression, purification, and reconstitution of 2-AqpZ and [10%^1^H,^13^C,^15^N]-AqpZ were almost the same as those of U-^13^C,^15^N-labeled AqpZ except for a few differences. 2-AqpZ was expressed using 2-^13^C-glycerol as the sole carbon source. [10%^1^H,^13^C,^15^N]-AqpZ was expressed with 10/90% H_2_O/D_2_O in M9 medium using ^15^NH_4_Cl as the sole nitrogen source and ^2^H,^13^C-glucose as the sole carbon source, which is commonly referred to as the reduced adjoining protonation labeling scheme (*31*). All purification and reconstitution steps were performed in the buffers containing 100% H_2_O. Next, the proteoliposome sample was incubated in the buffer containing 10/90% H_2_O/D_2_O to achieve 10% protonation of AqpZ in the exchangeable sites. Last, all protons in protein, including protons in the transmembrane domains, are ~90% diluted.

The proteoliposome sample of U-^13^C,^15^N-labeled AqpZ and 2-AqpZ containing 30% water content (100% H_2_O) (total weight of 45.0 mg) was packed into a thin-wall 3.2-mm rotor, and proteoliposome samples of [10%^1^H,^13^C,^15^N]-AqpZ containing 30% water content (10/90% H_2_O/D_2_O) (total weight of 9.0 mg) was packed into a 1.9-mm rotor for ssNMR experiments.

### ssNMR experiments

All ssNMR experiments were performed on a Bruker Avance III 800-MHz ssNMR spectrometer. All ^13^C-detected 3D DIPSHIFT and 3D CONCA experiments were conducted using a 3.2-mm triple-resonance (HCN) e-free probe. 3D ^1^H-detected hCANH, hCONH, and a series of 2D ^1^H_N_-^15^N CP-HSQC spectra for the measurements of R_1_ and R_1ρ_ were performed with a 1.9-mm quadra-resonance (HCND) probe at a MAS rate of 40 kHz. Sample temperatures of all experiments were calibrated using internal references of H_2_O and maintained at 283 K. The chemical shifts were referenced to adamantane used as an external reference (40.48 ppm for the methylene carbon) (*48*).

In 3D $R18_{1}^{7}$ DIPSHIFT experiments, an $R18_{1}^{7}$ module (*49*) was used for recoupling ^1^Hα-^13^Cα dipolar coupling in F2 dimension, and 2D NCA (F1-F3 dimension) was used to achieve site-specific resolution. Experiments were performed at a MAS rate of 10 kHz. $R18_{1}^{7}$ recoupling was implemented in a constant time manner with a ^1^H field strength of 9*ω_MAS_ (ω_MAS_ represents the MAS rate in radian per second). The evolution time of ^1^Hα-^13^Cα dipolar interactions is 0.6 ms. Z-filtered TEDOR (*50*) DIPSHIFT experiments were used for the measurement of S_NCα_ and performed at a MAS rate of 14 kHz. Dipolar recoupling between ^13^C and ^15^N was done using π-pulse trains, and two π pulses per rotor period are applied on the ^15^N channel. Twelve spectra were acquired with the following TEDOR dephasing times: 0.57, 1.14, 2.28, 3.42, 4.57, 5.71, 6.85, 8.00, 10.28, 12.57, 14.85, and 16.57 ms. ^15^N-R_1ρ_ measurements were done by the insertion of a ^15^N spinlock pulse with a field strength of 10 kHz after the ^1^H to ^15^N CP in the ^15^N-^1^H_N_ correlation spectrum. ^15^N spinlock pulse durations were set to 0, 10, 20, 30, 40, 50, 60, 70, 80, 100, 120, 140, 160, 180, 200, and 220 ms. ^15^N-R_1_ measurements were done by the insertion of ^15^N inversion recovery time after the ^1^H to ^15^N CP in the ^15^N-^1^H_N_ correlation spectrum. ^15^N inversion recovery times were set to 0, 3, 5, 8, 16, and 32 s. The details of parameter settings of ^1^H-detected 3D hCANH and hCONH experiments were described by Fricke *et al.* (*46*). The pulse sequences and the parameter details of ssNMR experiments are provided in figs. S1 and S5.

### Data analysis

#### *Analysis of SC*_α*H*α_

The dipolar order parameter (*S*, *S* = *D*_expt_/*D*_rigid_) is defined as the ratio between the experimentally determined dipolar couplings (*D*_expt_) and the rigid limit (*D*_rigid_) (*18*). The *D*_rigid_ for the ^1^H─^13^C bond was calculated using a ^1^Hα-^13^Cα one-bond length of 1.10 Å (*18*). Extracting the dipolar lineshapes of ^1^Hα-^13^Cα couplings by NMRPipe (*51*) and combining with the theoretical simulation using SIMPSON (*52*), we can get the exact size of site-specific *D*_expt_.

#### *Analysis of SNC*_α_

The *D*_rigid_ for the N─C bond was calculated using a ^15^N-^13^Cα one-bond length of 1.46 Å (*50*, *53*). For measurements of ^15^N-^13^Cα dipolar couplings, the curves of signal intensities extracted from z-filtered TEDOR experiments as a function of dephasing times were fitted using the equations described by Jaroniec *et al.* (*50*). The peak amplitudes were extracted using the software Sparky (*54*). The simulations were performed on a ^15^N_[*i*]_^13^C_[*i*]_^15^N_[*i* + 1]_ three-spin system and the *J*-coupling terms can be neglected due to the removal of ^13^C-^13^C *J*-coupling in ^13^C sparse labeling of 2-AqpZ. At these conditions, the ^15^N-^13^Cα z-filtered TEDOR trajectory was calculated as follows

$$S_{i}\left(t_{\text{mix}}\right)=\Lambda_{i}\left\{1-{\left[J_{0}\left(\sqrt{2}\;D_{i1}t_{\text{mix}}\right)\right]}^{2}\right\}\left\{1+{\left[J_{0}\left(\sqrt{2}\;D_{i2}t_{\text{mix}}\right)\right]}^{2}\right\}$$ (1)

and

$$\Lambda_{i}=\frac{1}{2^{N_{i}}}V_{i}\left(0\right)\lambda_{i}\text{exp}\left(-\Gamma_{i}t_{\text{mix}}\right)$$ (2)

where *i* represents the *i*th ^13^Cα nucleus, *N_i_* is the number of ^15^N spins simultaneously coupled to the *i*th ^13^Cα nucleus; here, *N_i_* = 2. *J*_0_(*x*) is a Bessel function of the zeroth order. *D*_*i*1_ and *D*_*i*2_ are the one-bond and two-bond dipolar couplings, respectively (expressed in hertz). *V_i_* (0) is an overall amplitude, and λ*_i_* is an overall amplitude scaling factor. Γ*_i_* is the transverse relaxation rate. Fitted parameters were *V_i_* (0), λ*_i_*, Γ*_i_*, *D*_*i*1_, and *D*_*i*2_. The fittings were performed based on the Matlab language environment.

#### Analysis of ^15^N-R_1_ρ and ^15^N-R_1_

In the data analysis by NMRPipe, a sine square (-pow 2) window function with a first point scaled to 0.167 (-c 1.0) was multiplied to the x, y, and z vectors. -g1 and -g2 of Lorentz-to-Gauss window function were set 40 and 80 Hz, respectively, to increase the resolution of the ^15^N-^1^H_N_ correlation spectrum. Peak amplitudes [*M*(*t*)] extracted from ^15^N-^1^H_N_ correlation spectra as a function of spinlock times (*t*) were fitted using the single exponential function

$$M\left(t\right)=M_{0}\;e^{-t\times R\text{1ρ}}$$ (3)

Peak amplitudes [*M*(*t*)] extracted from ^15^N-^1^H_N_ correlation spectra as a function of inversion recovery times (*t*) were fitted using the single exponential function

$$M\left(t\right)=M_{0}\left(1-2\times e^{-t\times R1}\right)$$ (4)

The fitted parameters were *M*_0_ and *R*_1ρ_ and *R*_1_. The peak amplitudes were extracted using the software Sparky (*54*). The fittings were performed based on the Matlab language environment.

### The 3D GAF model calculation

In the 3D GAF model, the collective motion of each secondary structure segment along three orthogonal axes (α, β, and γ) and with different amplitudes (σ_α_, σ_β_, and σ_γ_) is approximated as a rigid-body motion with a single timescale (τ_c_) (*28*). The parameters σ_α_, σ_β_, σ_γ_, and τ_c_ depend on experimentally measured *S*_CαHα_, ^15^*N-R*_1_, and ^15^*N-R*_1ρ_, and these parameters can be obtained by screening these four parameters to make the theoretically calculated *S*_CαHα_, ^15^*N-R*_1_, and ^15^*N-R*_1ρ_ to fit the experimentally measured ones. In this model, additional internal fast motions on the ps-to-ns timescales can be neglected because they are highly restricted as evidence of high dipolar *S*_CαHα_s and *S*_NCα_s and such small ^5^*N-R*_1_s observed in AqpZ.

For 3D GAF motion, the squared order parameters $S_{\mu \nu }^{2}$ (3D GAF-S^2^) can be expressed as follows (*28*)

$$\begin{array}{c} S_{\mu \nu }^{2}=\frac{4\pi }{5}\sum_{l,k,k\prime ,m,m\prime =-2}^{2}{\left(-i\right)}^{k-k\prime }\text{exp}\left\{-\frac{\sigma_{\alpha }^{2}\left(k^{2}+k\prime 2\right)}{2}-\sigma_{\beta }^{2}l^{2}-\frac{\sigma_{\gamma }^{2}\left(m^{2}+m\prime 2\right)}{2}\right\}\times \\ d_{\mathrm{kl}}^{\left(2\right)}\left(\frac{\pi }{2}\right)d_{k\prime l}^{\left(2\right)}\left(\frac{\pi }{2}\right)d_{\mathrm{mk}}^{\left(2\right)}\left(\frac{\pi }{2}\right)d_{m\prime k\prime }^{\left(2\right)}\left(\frac{\pi }{2}\right)\;Y_{2m}\left(e_{\mu }^{\mathrm{pp}}\right)\;Y_{2m\prime }^{*}\left(e_{\nu }^{\mathrm{pp}}\right) \end{array}$$ (5)

where σ_α_, σ_β_, and σ_γ_ are amplitudes of fluctuations against the respective axes of motion (expressed in radians); $e_{\mu }^{\mathrm{pp}}$ = (θ_μ_,φ_μ_) and $e_{\nu }^{\mathrm{pp}}$ = (θ_v_,φ_v_) define the symmetry axis for interaction μ and ν (for auto relaxation μ = ν; here, θ and φ are essentially spherical coordinates for the appropriate bond vectors) in the α, β, and γ frame rigidly attached to molecular fragment (*pp*); $d_{\mathrm{kl}}^{\left(2\right)}$(π/2) are the reduced Wigner matrix elements; and *Y*_2m_ are the second spherical harmonics.

The timescales of the 3D GAF model were obtained based on the framework of the SMF model (*55*). In the SMF model, the ^15^*N-R*_1ρ_ is mainly modulated by ^15^N CSA and ^15^N-^1^H dipole coupling interaction. The theoretical description of ^15^*N-R*_1ρ_ can be expressed as follows

$$R_{1}=\frac{1}{T_{1}}=R_{1}^{\text{CSA}}+R_{1}^{\text{NH}}$$ (6)

$$R_{1\rho }=\frac{1}{T_{1\rho }}=\frac{1}{2}R_{1}+R_{1\rho }^{\text{CSA}}+R_{1\rho }^{\text{NH}}$$ (7)

where $R_{1}^{\mathrm{CSA}}$ and $R_{1}^{\mathrm{NH}}$ are the longitudinal relaxation rates resulting from the CSA and dipolar interaction, and $R_{1\rho }^{\text{CSA}}$ and $R_{1\rho }^{\text{NH}}$ are the additional dependence on the transverse relaxation rate. Approximating the CSA tensor as an axially symmetric item, these contributions can be expressed as follows

$$R_{1}^{\text{CSA}}=\frac{3}{4}{\left(\delta_{\text{CSA}}\omega_{N}\right)}^{2}J\left(\omega_{N}\right)$$ (8)

$$R_{1}^{\text{NH}}=\frac{\delta_{\text{NH}}^{2}}{4}[J\left(\omega_{H}-\omega_{N}\right)+3J\left(\omega_{N}\right)+6J\left(\omega_{H}+\omega_{N}\right)]$$ (9)

$$\begin{array}{c} R_{1\rho }^{\text{CSA}}=\frac{{\left(\delta_{\text{CSA}}\omega_{N}\right)}^{2}}{4}[\frac{1}{3}J\left(\omega_{1}-2\omega_{r}\right)+\frac{2}{3}J\left(\omega_{1}-\omega_{r}\right)\\ +\frac{2}{3}J\left(\omega_{1}+\omega_{r}\right)+\frac{1}{3}J\left(\omega_{1}+2\omega_{r}\right)] \end{array}$$ (10)

$$\begin{array}{c} \;R_{1\rho }^{\text{NH}}=\frac{\delta_{\text{NH}}^{2}}{4}[3J\left(\omega_{H}\right)+\frac{1}{3}J\left(\omega_{1}-2\omega_{r}\right)+\frac{2}{3}J\left(\omega_{1}-\omega_{r}\right)\\ +\frac{2}{3}J\left(\omega_{1}+\omega_{r}\right)+\frac{1}{3}J\left(\omega_{1}+2\omega_{r}\right)] \end{array}$$ (11)

where ω_1_ is the spinlock power expressed in radians per second; ω_H_ and ω_N_ are the Larmor frequencies of ^1^H and ^15^N expressed in radians per second; ω_r_ is the MAS rate expressed in radians per second; δ_NH_ is the strength of the N-H dipolar coupling; and δ_CSA_ is the reduced CSA of ^15^N. *J*_solid_(ω) is the spectral density function in a solid sample

$$J_{\text{solid}}\left(\omega \right)=\frac{2}{5}\left(1-S^{2}\right)\frac{\tau_{c}}{1+{\left(\omega \tau_{c}\right)}^{2}}$$ (12)

where *S*^2^ is the square of the HN-dipolar order parameter, and τ_c_ is the effective correlation time. Substituting *S*^2^ in *J*(ω) with 3D GAF-S^2^, we can calculate *R*_1_ and *R*_1ρ_ modulated by 3D GAF. In this work, we adjusted six parameters σ_α_, σ_β_, σ_γ_, τ_c_, δ_θ_, and δ_ϕ_ (δ_θ_ and δ_ϕ_ describe the transformation between the molecular and 3D GAF frame, which is not known a priori) to make the theoretically calculated *S*_CαHα_, ^15^*N-R*_1_, and ^15^*N-R*_1ρ_ fitted to the experimentally measured ones simultaneously in Matlab. The quality of the fitting was evaluated with the reduced χ^2^ (χ_red_^2^), which is expressed as follows

$$\begin{array}{c} \chi_{\text{red}}^{2}=\frac{1}{N1+N2+N3-p-1}\{\sum_{1}^{N1}[\frac{{\left(S_{C\alpha H\alpha ,i,\text{exp}}^{2}-S_{C\alpha H\alpha ,i,\text{cal}}^{2}\right)}^{2}}{\sigma_{C\alpha H\alpha ,i,\text{exp}}^{2}}]\\ +\sum_{1}^{N2}[\frac{{\left(R_{1\rho ,i,\text{exp}}-R_{1\rho ,i,\text{cal}}\right)}^{2}}{\sigma_{1\rho ,i,\text{exp}}^{2}}]+\sum_{1}^{N3}[\frac{{\left(R_{1,i,\text{exp}}-R_{1,i,\text{cal}}\right)}^{2}}{\sigma_{1,i,\text{exp}}^{2}}]\} \end{array}$$ (13)

where *N*1, *N*2, and *N*3 are the numbers of available experimental points for *S*_CαHα_, ^15^*N-R*_1_, and ^15^*N-R*_1ρ_, respectively. (*N*1 + *N*2 + *N*3 − *p* − 1) denotes the degrees of freedom in which *p* = 6. σ_CαHα_, σ_1_, and σ_1ρ_ are the experimental errors for *S*_CαHα_, ^15^*N-R*_1_, and ^15^*N-R*_1ρ_, respectively. When the reduced χ^2^ is close to 1, the ACMs account well for the experimentally measured dipolar order parameters and relaxation rates. The model calculations were performed based on the Matlab language environment.

### MD simulations

The structure of AqpZ is built based on the high-resolution structure of AqpZ reconstituted in synthetic bilayers, previously reported by our group (*30*). The membrane bilayer is composed of POPC and POPG. The ratio of POPC to POPG is 3:1, and the total number of lipids is 800. The membrane bilayer model and protein insertion are accomplished by the CHARMM-GUI web server (*56*). The CHARMM36 force fields were used for proteins and membranes (*57*), and the TIP3P model (*58*) was used for water molecules. The systems were energy-minimized for 5000 steps using the steepest descent method. The proteins and lipids were restrained with 10 and 2.5 kJ/mol·Å^2^ force constants in the minimization steps, respectively. NVT simulations (500 ps) with position constraints applied on protein-heavy atoms and lipids were performed. Then, 1-ns NPT simulations with gradually decreased position constraints on the protein and lipid heavy atoms were performed to equilibrate the systems. The length of bonds relative to hydrogen atoms was constrained with the linear constraint solver algorithm (*59*). A semi-isotropic scheme was used to couple the lateral and perpendicular pressures separately. The particle-mesh Ewald method (*60*) was used to calculate long-range electrostatics with a cutoff of 12 Å. The temperature was kept at 300 K with the V-rescale method, and the pressure was coupled by a Parrinello-Rahman barostat. The production runs were performed with a 2-fs time step by the CUDA version of the AMBER suite (*61*).
