# Supplementary material for: Fast collective motions of backbone in transmembrane α helices are critical to water transfer of aquaporin
Source: Sci Adv. 2024 May 8;10(19):eade9520. doi: 10.1126/sciadv.ade9520 (PMC11078191; doi:10.1126/sciadv.ade9520)
Supplement: Supplementary file 1 — Figs. S1 to S17 Tables S1 to S3 [file sciadv.ade9520_sm.pdf]

Science Advances
AAAS

Supplementary Materials for

Fast collective motions of backbone in transmembrane α helices are critical to water transfer of aquaporin

Huan Tan *et al.*

Corresponding author: Mojie Duan, mjduan@wipm.ac.cn; Jun Yang, yangjun@wipm.ac.cn

Sci. Adv. **10**, eade9520 (2024)
DOI: 10.1126/sciadv.ade9520

This PDF file includes:

Figs. S1 to S17
Tables S1 to S3

Supplementary Figures

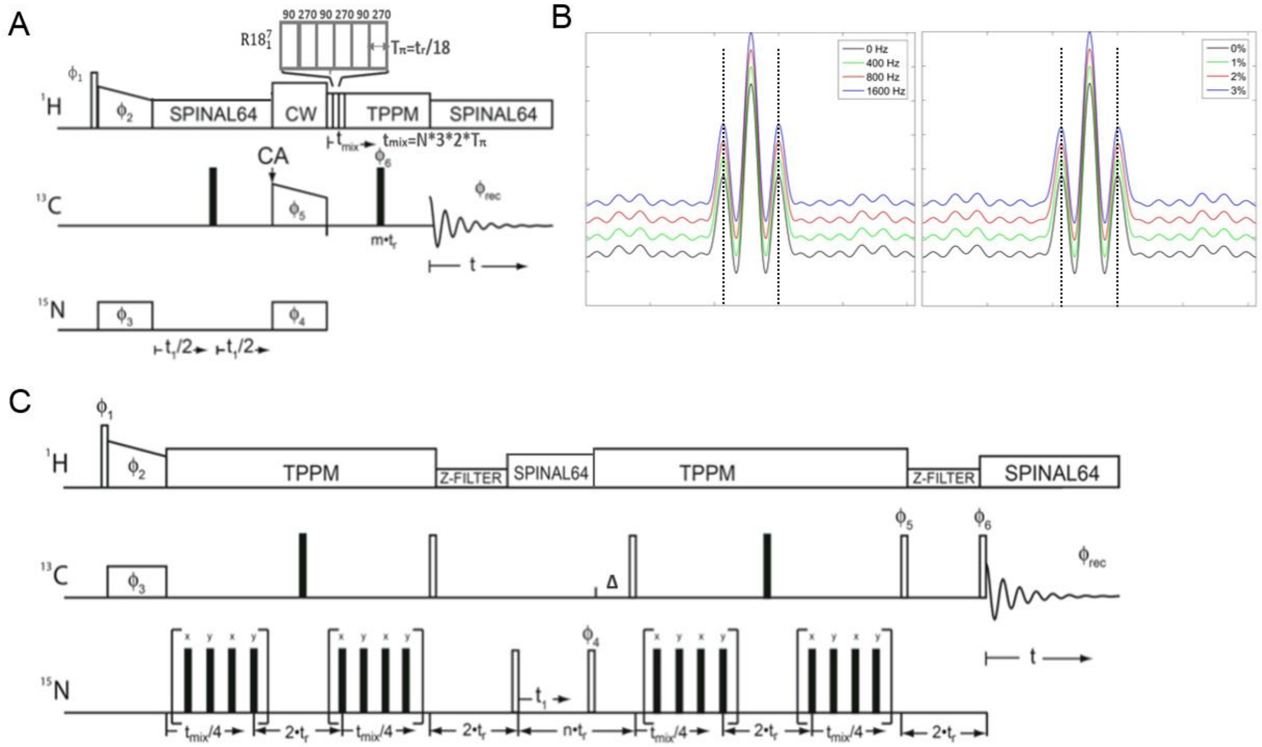

Fig. S1. Pulse sequences of 3D DIPSHIFT experiments for measuring $^{1}H\alpha$-$^{13}C\alpha$ (A) and ^{15}N-$^{13}C\alpha$ (C) dipolar couplings.

(**A**) $R18_1^7$ recoupling was implemented in a constant-time manner (mt_r duration) with ^{1}H field strength of $9*\omega_{MAS}$ (ω_{MAS} =10 kHz). The duration of 180° pulses (T_π) of the $R18_1^7$ pulse train is one-eighteenth of a rotor cycle ($T_\pi=t_r/18$). The duration of $R18_1^7$ pulse train is t_{mix}, followed by TPPM decoupling with a ^{1}H field strength of 90 kHz during the remainder of the echo period (mt_r-t_{mix}). SPINAL64 decoupling with a ^{1}H field strength of 65 kHz is applied during t_1 and t_{rec} evolution. Typical 90° pulse lengths of 3.0 μs (^{1}H), 4.3 μs (^{13}C), and 7.0 μs (^{15}N) were used. In (**A**) every sampling interval during t_{mix} is $6*T_\pi$ and 2D NCA spectra are recorded as a function of t_{mix}, with the following phases: φ_1=y -y, φ_2=x, φ_3=x, φ_4=x, φ_5=x x y y -x -x -y -y, φ_6=x x y y -x -x -y -y, φ_{rec}=x -x y -y -x x -y y. All pulses without phases indicated are applied along x.

(**B**) The sensitivity of the HC-$R18_1^7$ dipole recoupling module to the radiofrequency (RF) frequency offset (left) and field stability (right) in the proton channel by Simpson theoretical simulations. The frequency offset of the H-RF field does not noticeably affect the HC-$R18_1^7$ dipole lineshape within the chemical shift anisotropy (CSA) range of protons (^{C}H), and the power fluctuation within 3% also does not noticeably affect the HC-$R18_1^7$ dipole lineshape within the ^{C}H CSA range.

(**C**) The 3D z-filtered TEDOR pulse sequence is used for ^{15}N-^{13}C dipolar recoupling and magnetization transferring based on the rotor synchronized π-pulse trains, and two π pulses per rotor period are applied on the ^{15}N channel with phase cycled xy-4. TPPM decoupling with a ^{1}H field strength of 90 kHz is applied during REDOR periods. SPINAL64 decoupling with a ^{1}H field strength of 65 kHz is applied during t_1 and t_{rec} evolution. Typical 90° pulse lengths of 3.0 μs (^{1}H), 4.3 μs (^{13}C), and 7.0 μs (^{15}N) were used. The delay Δ is calculated for each t_1 interval such that the time between the first and second REDOR periods is equal to an integer number of rotor

cycles. ^{1}H field strength of $1*\omega_{MAS}$ is applied to two Z-FILTER periods, which eliminates multiple-quantum and anti-phase spin coherences generated by ^{13}C-^{13}C J-coupling. The phases of all pulses are given as φ_1=16×(x) 16×(-x), φ_2=x, φ_3=x, φ_4=x -x, φ_5=y y -y -y, φ_6=x x x x y y y y -x -x -x -x -y -y -y -y, φ_{rec}=-y y y -y x –x -x x, y -y -y y -x x x -x, y -y -y y -x x x -x, -y y y -y x -x -x x and all remaining pulses are of phase x.

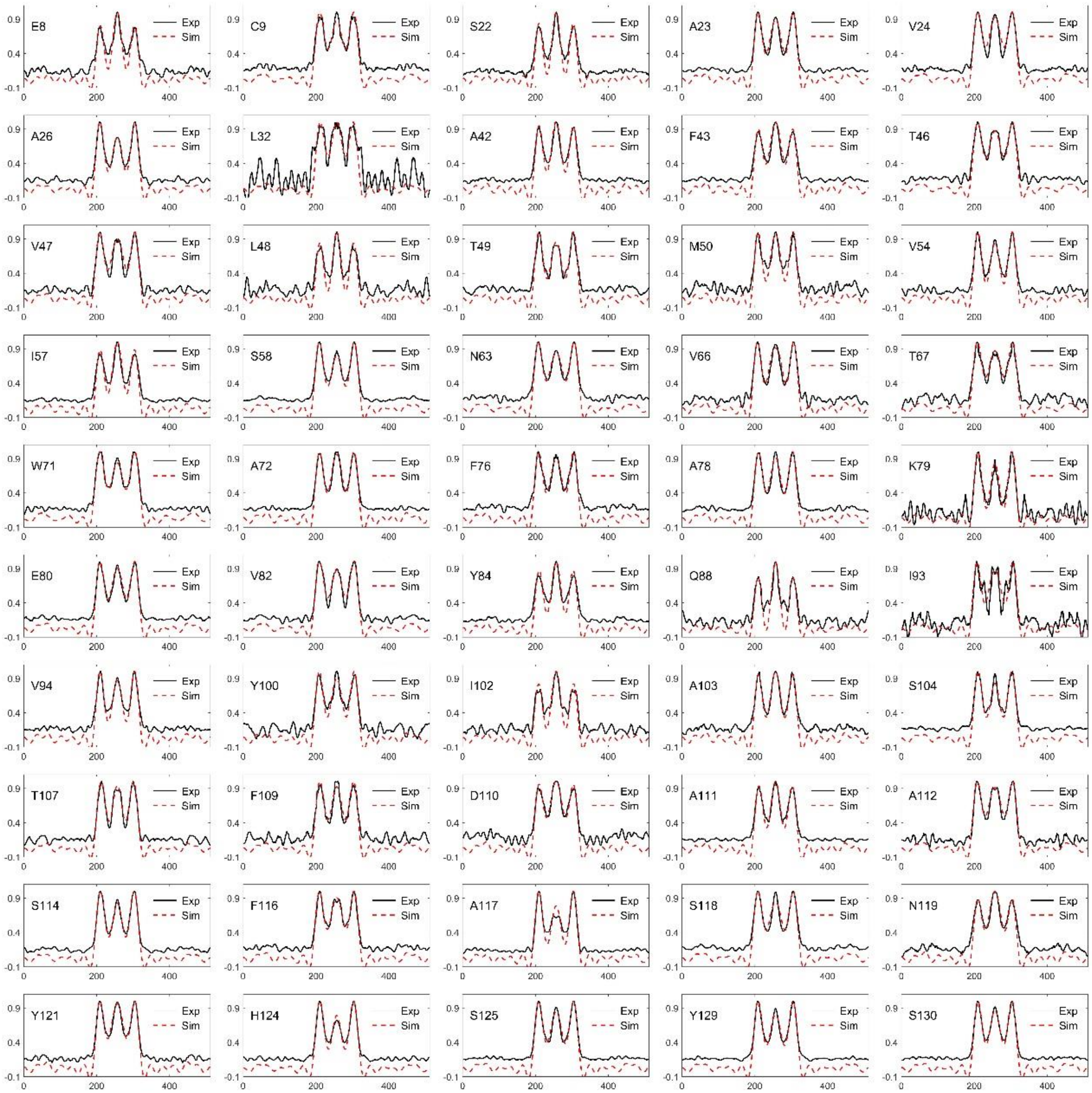

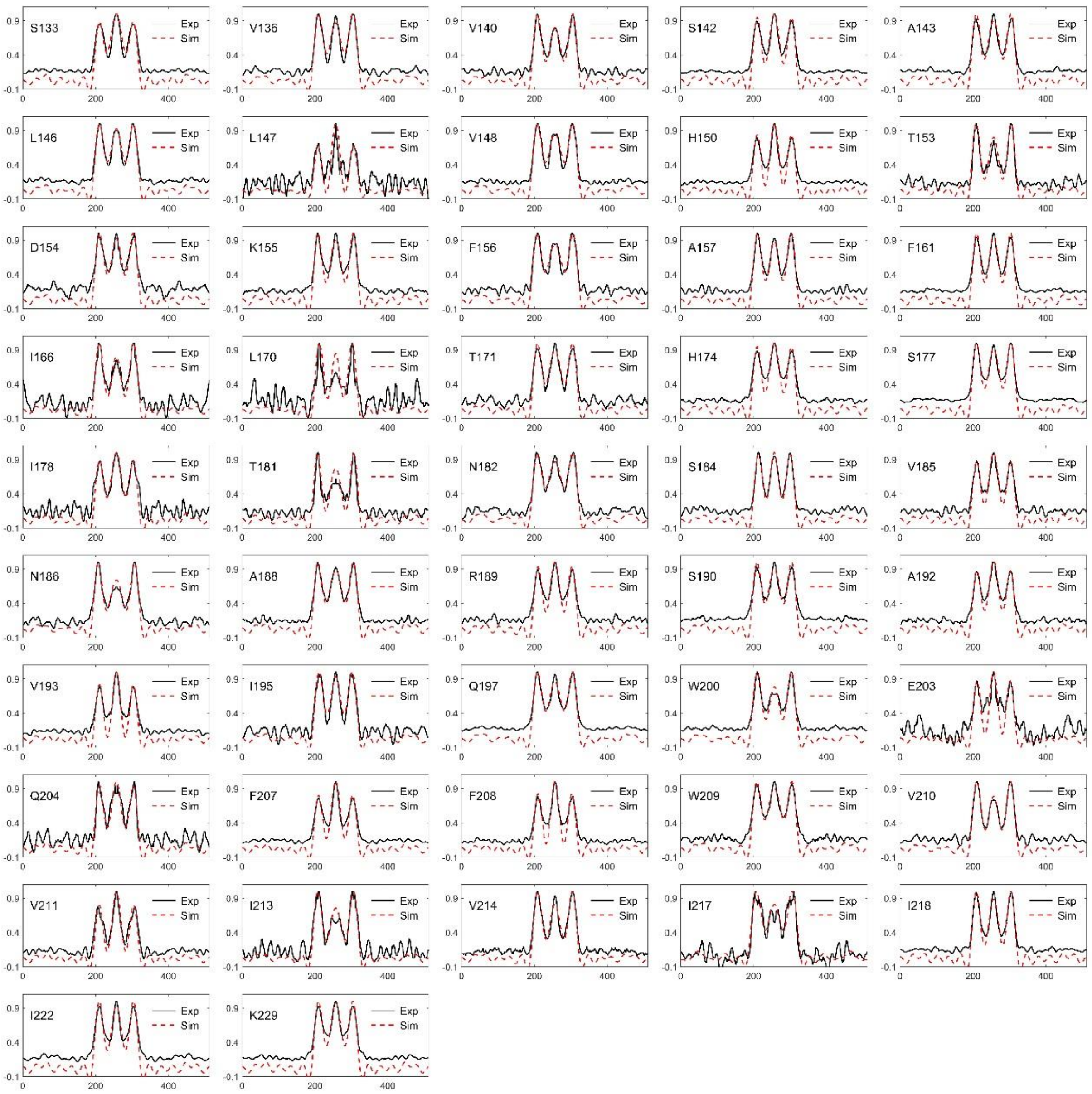

Fig. S2. Lineshapes of $^{1}H\alpha$-$^{13}C\alpha$ dipolar couplings extracted from 3D DIPSHIFT spectra. The experimental dipolar lineshapes and the corresponding best fittings were shown by black solid lines and red dashed lines, respectively.

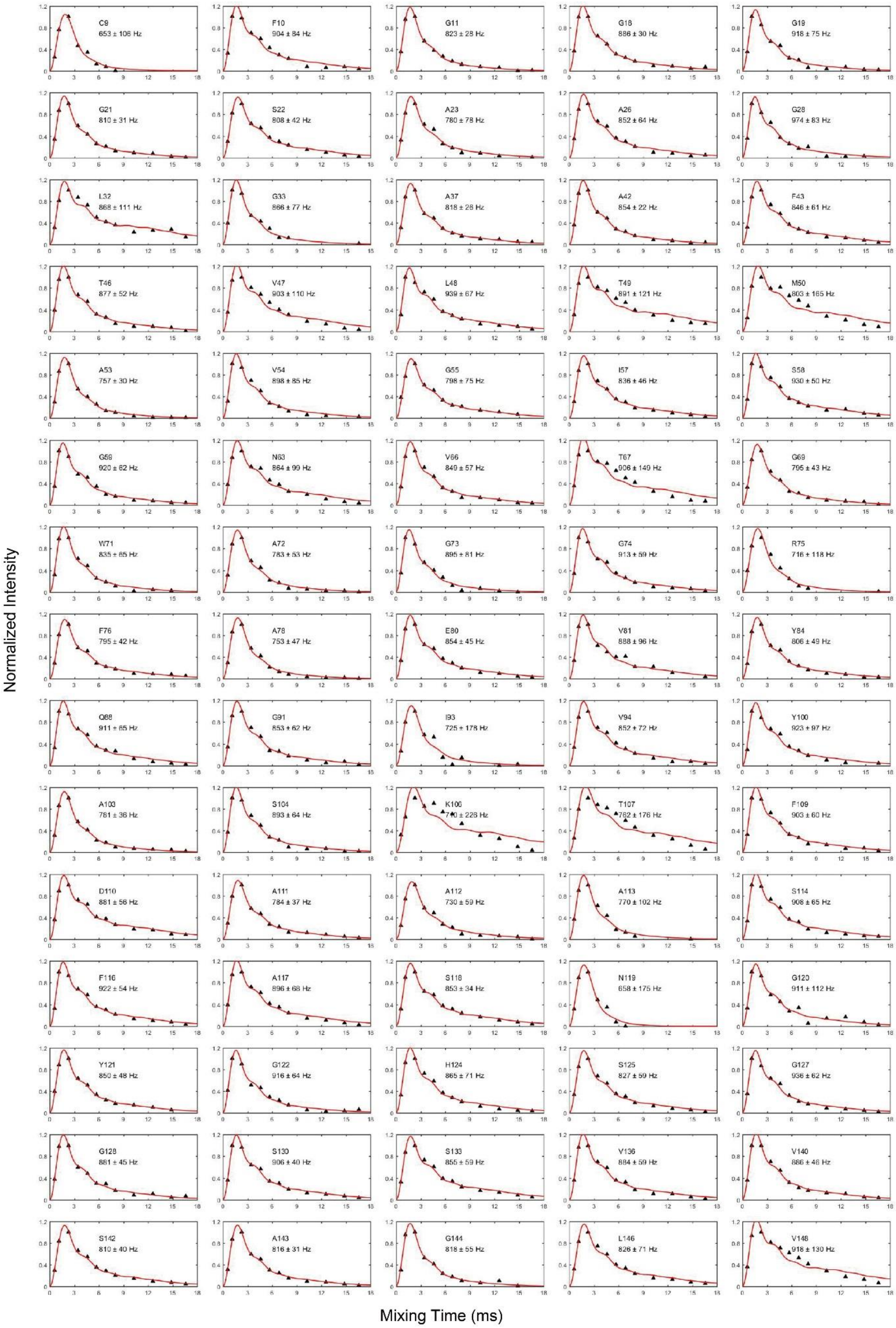

Normalized Intensity
Mixing Time (ms)
C9
653 ± 106 Hz
F10
904 ± 84 Hz
G11
823 ± 28 Hz
G18
886 ± 30 Hz
G19
918 ± 75 Hz
G21
810 ± 31 Hz
S22
808 ± 42 Hz
A23
780 ± 78 Hz
A26
852 ± 64 Hz
G28
974 ± 83 Hz
L32
868 ± 111 Hz
G33
866 ± 77 Hz
A37
818 ± 28 Hz
A42
854 ± 22 Hz
F43
846 ± 61 Hz
T46
877 ± 52 Hz
V47
903 ± 110 Hz
L48
939 ± 67 Hz
T49
891 ± 121 Hz
M50
803 ± 165 Hz
A53
757 ± 30 Hz
V54
898 ± 85 Hz
G55
798 ± 75 Hz
I57
836 ± 46 Hz
S58
930 ± 50 Hz
G59
920 ± 62 Hz
N63
864 ± 99 Hz
V66
849 ± 57 Hz
T67
906 ± 149 Hz
G69
795 ± 43 Hz
W71
835 ± 65 Hz
A72
783 ± 53 Hz
G73
895 ± 81 Hz
G74
913 ± 59 Hz
R75
716 ± 118 Hz
F76
795 ± 42 Hz
A78
753 ± 47 Hz
E80
854 ± 45 Hz
V81
888 ± 96 Hz
Y84
806 ± 49 Hz
Q88
911 ± 65 Hz
G91
853 ± 62 Hz
I93
725 ± 178 Hz
V94
852 ± 72 Hz
Y100
923 ± 97 Hz
A103
781 ± 36 Hz
S104
893 ± 64 Hz
K106
740 ± 228 Hz
T107
762 ± 176 Hz
F109
903 ± 60 Hz
D110
881 ± 56 Hz
A111
784 ± 37 Hz
A112
730 ± 59 Hz
A113
770 ± 102 Hz
S114
908 ± 65 Hz
F116
922 ± 54 Hz
A117
896 ± 68 Hz
S118
853 ± 34 Hz
N119
658 ± 175 Hz
G120
911 ± 112 Hz
Y121
850 ± 48 Hz
G122
916 ± 64 Hz
H124
865 ± 71 Hz
S125
827 ± 59 Hz
G127
936 ± 62 Hz
G128
881 ± 45 Hz
S130
906 ± 40 Hz
S133
855 ± 59 Hz
V136
884 ± 59 Hz
V140
886 ± 46 Hz
S142
810 ± 40 Hz
A143
816 ± 31 Hz
G144
818 ± 55 Hz
L146
826 ± 71 Hz
V148
918 ± 130 Hz

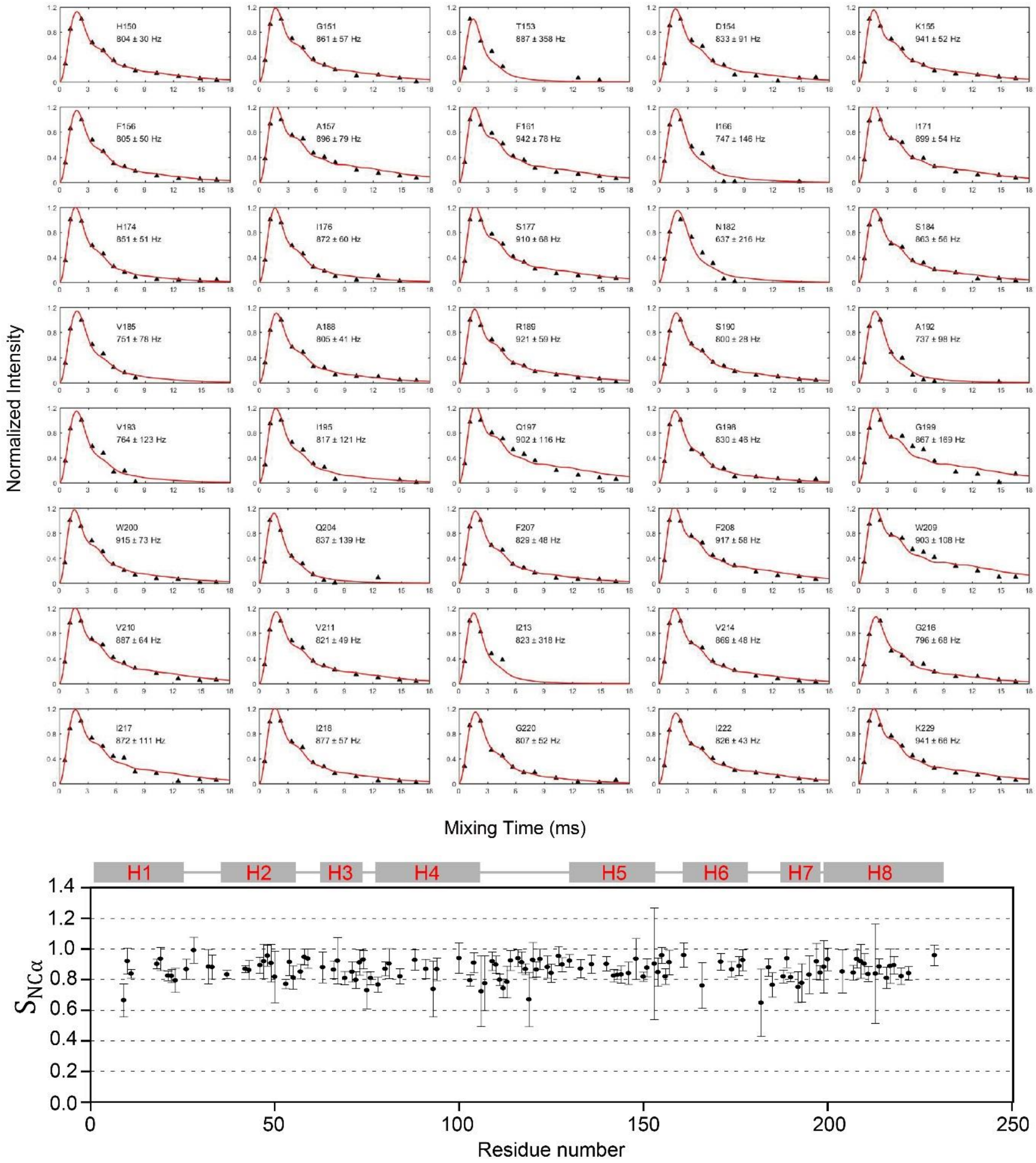

Fig. S3. One-bond ^{15}N-^{13}Cα dipolar order parameters of AqpZ. Above: experimental buildup curves (black triangle) of all residues extracted from 3D z-filtered TEDOR spectra of AqpZ, and the best fitting results for measuring ^{15}N-^{13}Cα couplings are shown in red lines. Below: one-bond ^{15}N-^{13}Cα dipolar order parameters as a function of residue numbers. The uncertainties of dipolar order parameters were estimated by a 95% confidence level interval. All one-bond dipolar order parameters were listed in **Table S2**.

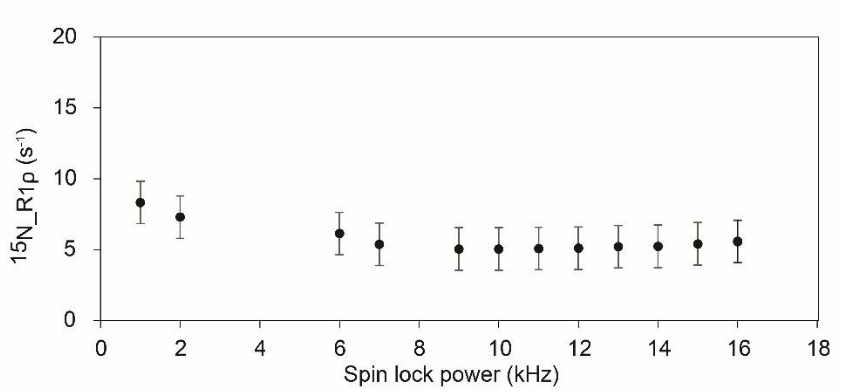

Fig. S4. Determination of the optimal ^{15}N spinlock power for site-specific $R_{1\rho}$ measurements. Bulk amide ^{15}N-$R_{1\rho}$s of 90% proton diluted AqpZ as a function of the spinlock field strength at a MAS rate of 40 kHz. To confirm that the fast MAS rate and proton dilution sufficiently suppress coherent contributions and result in notably small relaxation rates, we initially measured bulk ^{15}N-$R_{1\rho}$ at a series of spinlock power. The bulk ^{15}N-$R_{1\rho}$ of AqpZ reaches a plateau at spinlock power higher than 10 kHz, indicating that coherent effects (such as dipole interaction) to the ^{15}N-$R_{1\rho}$ are completely suppressed, in agreement with the results of studies on ASR (*18*).

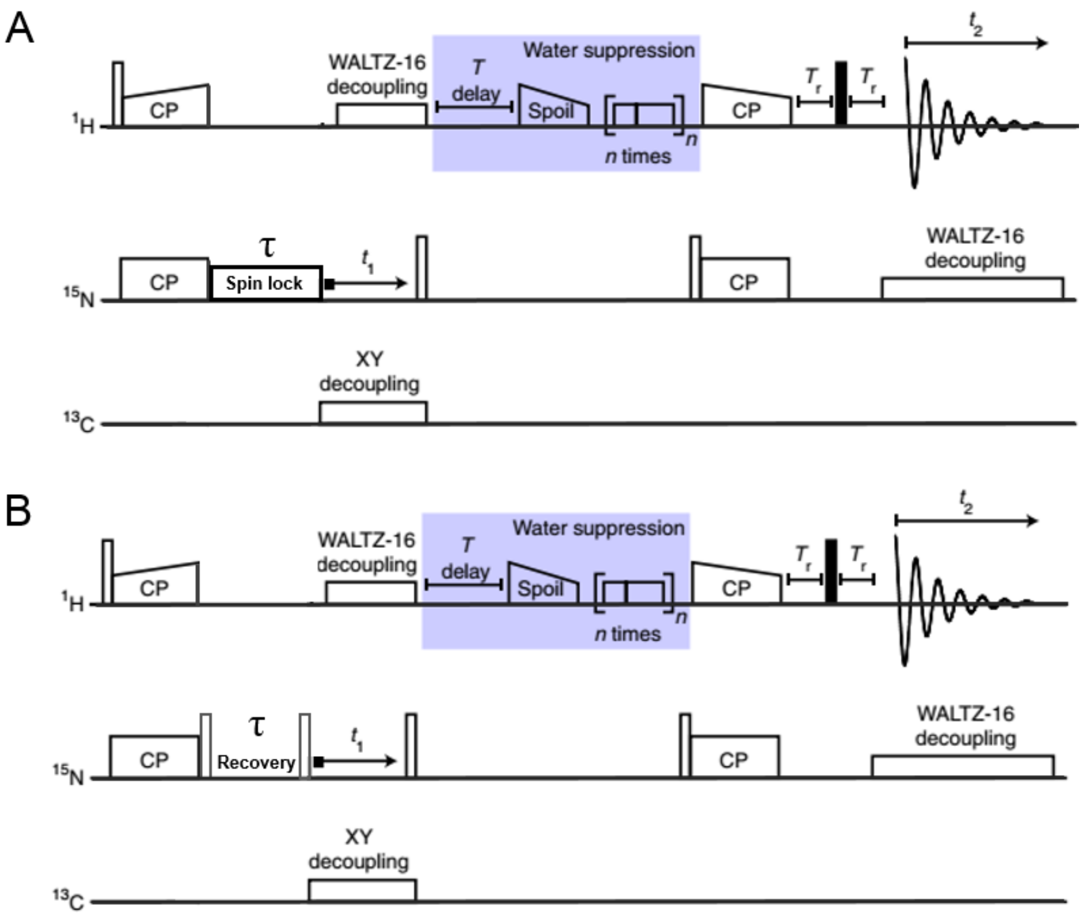

Fig. S5. Pulse sequence for ^{15}N-$R_{1\rho}$ (A) and ^{15}N-R_1 (B) measurements. ^{15}N-$R_{1\rho}$s are measured by inserting a ^{15}N spin-lock module after the ^{1}H/^{15}N cross-polarization (CP) in the ^{15}N-$^{1}H_N$ correlation spectrum at MAS rate of 40 kHz. ^{15}N-R_1s measurements were done by the insertion of ^{15}N inversion recovery time after the ^{1}H to ^{15}N CP in the ^{15}N-$^{1}H_N$ correlation spectrum at MAS rate of 40 kHz. Hollow and filled bars represent 90° and 180° pulses. ^{1}H /^{15}N CP contact time is

1000 μs with ^{1}H radio frequency (rf) field strength of about 70 kHz and ^{15}N rf field strength of about 30 kHz to satisfy the Hartmann–Hahn condition. The field strength of $^{5}N/^{1}H$ CP is almost the same as ^{1}H /^{15}N CP, but $^{15}N/^{1}H$ CP contact time is set to be 0.2 ms insuring one-bond polarization transfer. A linear ramp from 80 to 100% is set on the ^{1}H channel. All WALTZ-16 decoupling power is set to be 10 kHz (0.25*ω_{MAS}). XY decoupling power is set to 50 kHz. T delay for water suppression is set to 44 ms. The duration of the ramped water-suppression spoil pulse is set to 1.2 ms and its power is the same as the first ^{1}H CP pulse. The power level of the water suppression pulse train is set to 11 kHz and the pulse durations are set to 20 ms and 34 ms. Typical 90° pulse lengths of 2.2 μs (^{1}H), 4.5 μs (^{13}C), and 5.5 μs (^{15}N) were used.

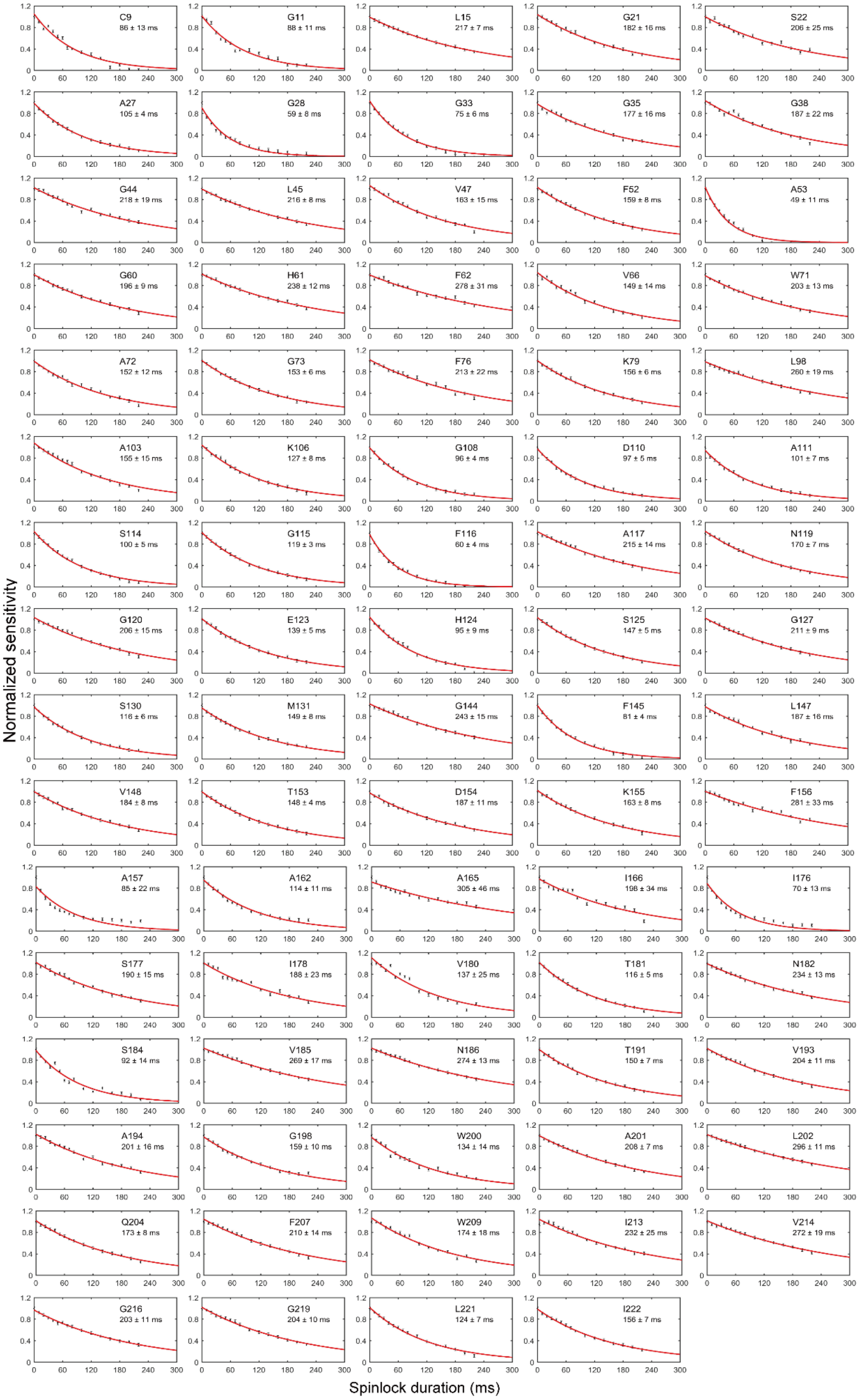

Fig. S6. Signal intensity trajectories extracted from a series of ^{15}N-$^{1}H_N$ correlation spectra of AqpZ (black dots) and the best fitting results (red lines) of $R_{1\rho}$ measurements.

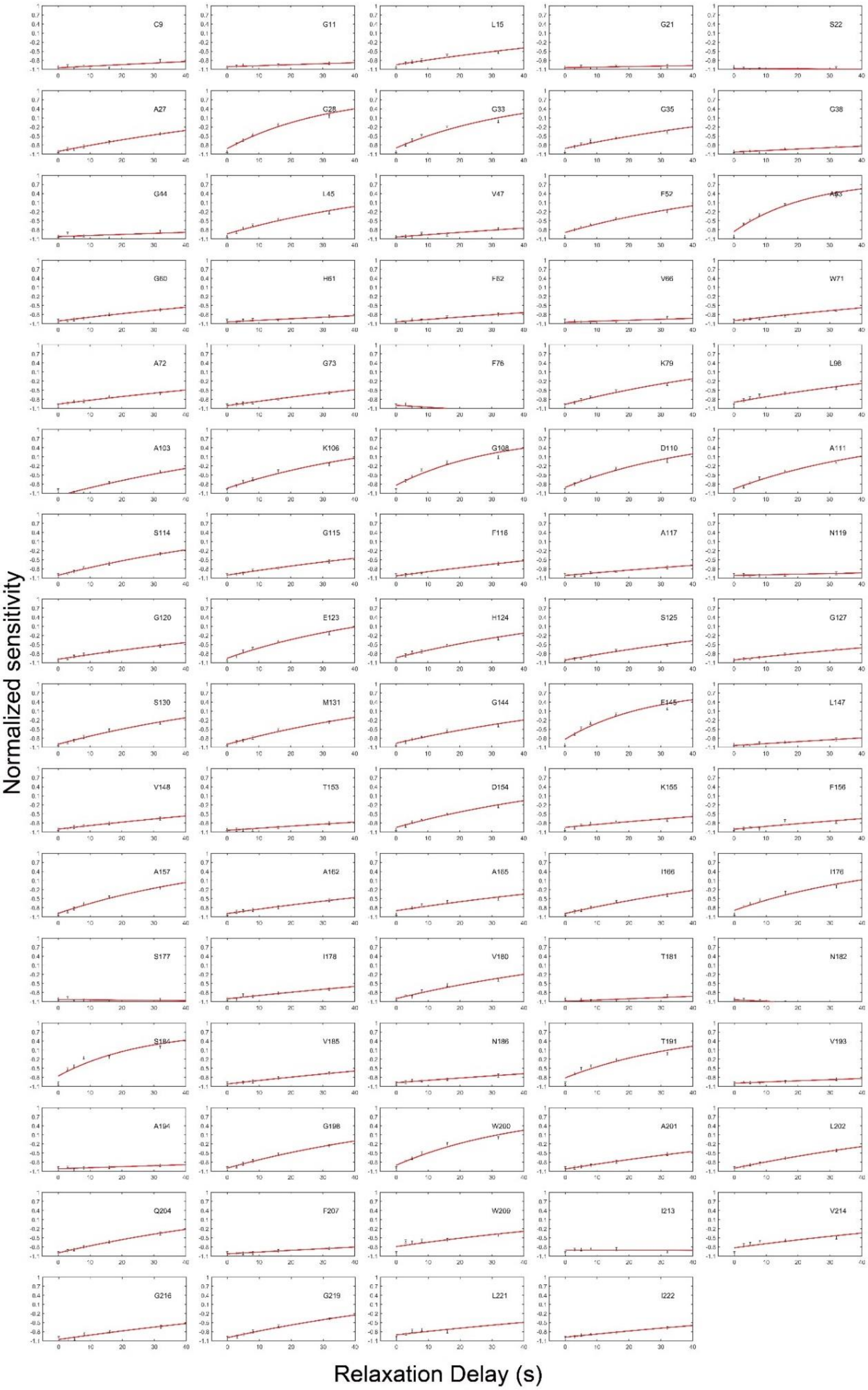

Fig. S7. Signal intensity trajectories extracted from a series of ^{15}N-$^{1}H_N$ correlation spectra of AqpZ (black dots) and the best fitting results (red lines) of R_1 measurements.

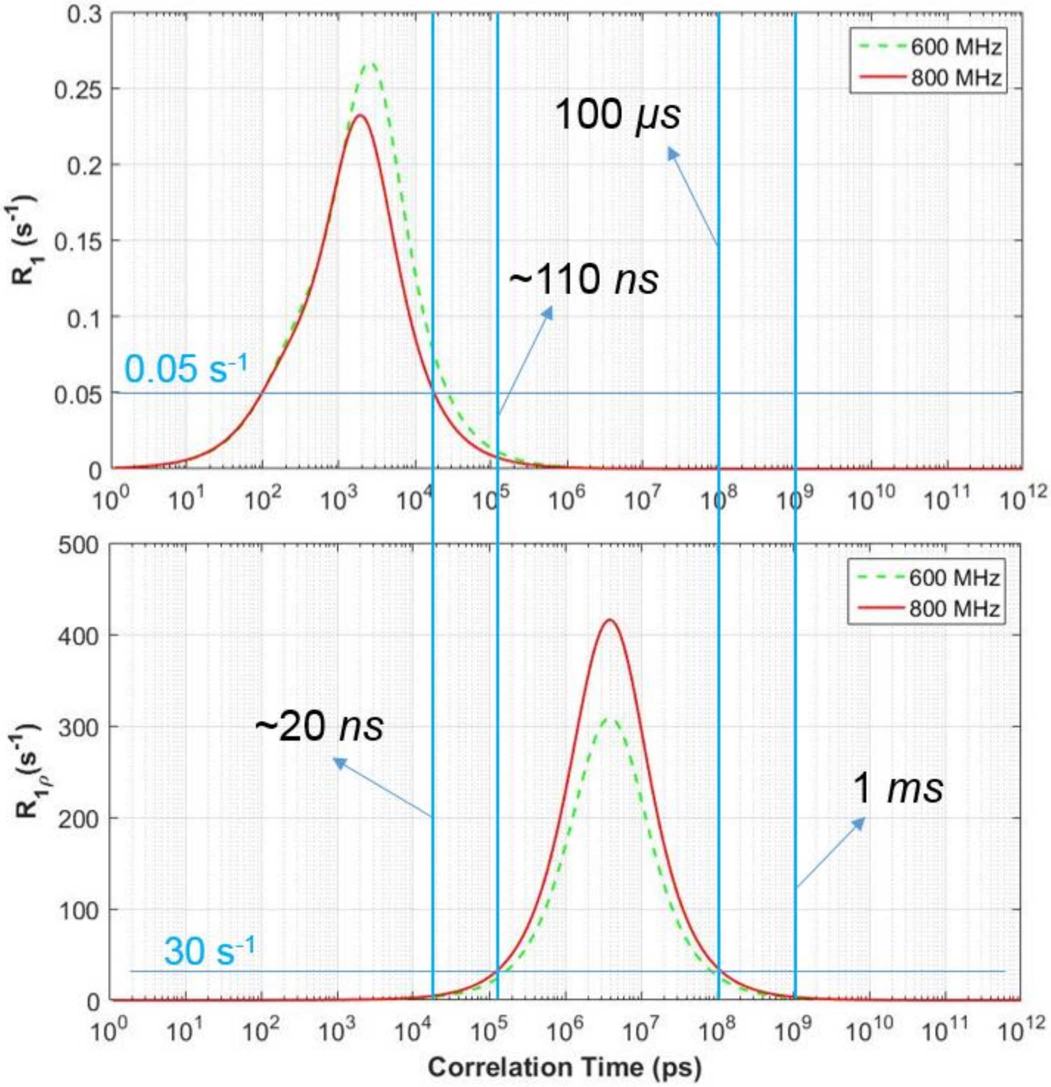

Fig. S8. Theoretical ^{15}N-R$_1$ and ^{15}N-R$_{1\rho}$ were calculated using Simple Model Free approach as a function of correlation time. The calculation was performed at field corresponding to ^{1}H Larmor frequencies of 800 MHz (red solid line) and 600 MHz (green dashed line) for a typical S^2_{NH} of 0.9. The ^{1}H-^{15}N one bond length was set 1.02 Å. The effective spin-lock power was set 10 kHz. The MAS rate was set 40 kHz and the reduced chemical shift anisotropy of ^{15}N was set 170 ppm. The aforementioned conditions are consistent with the experimental conditions of our work.

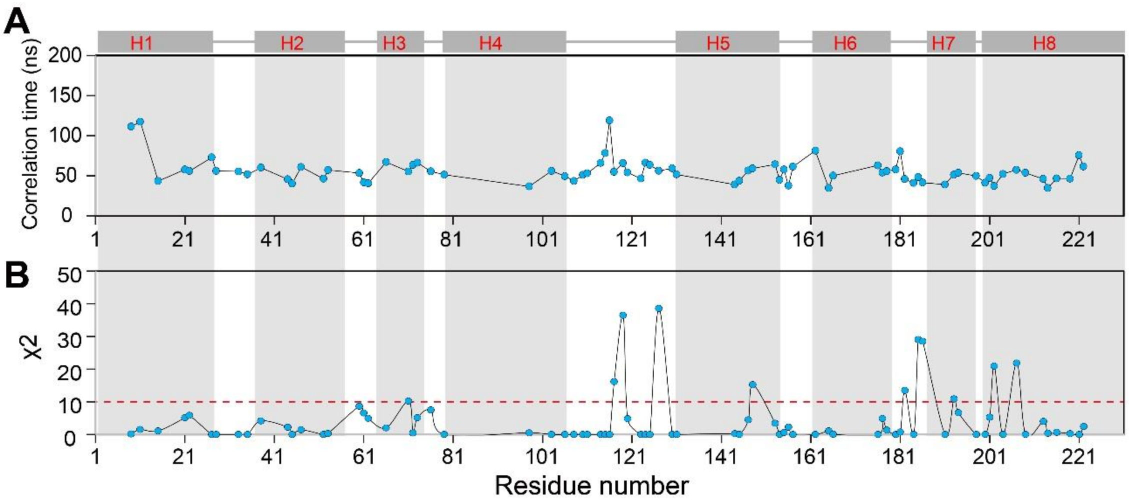

Fig. S9. The dominant correlation time of local motions within AqpZ calculated by Simple Model Free approach as a function of residue numbers. The calculation was performed at field corresponding to ^{1}H Larmor frequencies of 800 MHz. The ^{1}H-^{15}N one bond length was set 1.02 Å. The effective spin-lock power was set 10 kHz. The MAS rate was set 40 kHz and the reduced chemical shift anisotropy of ^{15}N was set 170 ppm. The aforementioned conditions are consistent with the experimental conditions of our work. The range of the time scale is set from 1 ps to 1 ms, within which both ^{15}N-R_1 and ^{15}N-$R_{1\rho}$ exhibit sensitivity, to explore the dominant motion time scale. Most residues within the helices of AqpZ exhibit χ^2 values below 10, which confirms the reliability of the dominant motion time scale calculated by the SMF model.

In the SMF model, the ^{15}N-R_1 and ^{15}N-$R_{1\rho}$ is mainly modulated by ^{15}N chemical shift anisotropy (CSA) and ^{15}N-^{1}H dipole coupling interaction. The theoretical description of ^{15}N-$R_{1\rho}$ can be expressed as follows：

$$R_1 = \frac{1}{T_1} = R_1^{CSA} + R_1^{NH} \quad \text{(S1)}$$

$$R_{1\rho} = \frac{1}{T_{1\rho}} = \frac{1}{2}R_1 + R_{1\rho}^{CSA} + R_{1\rho}^{NH} \quad \text{(S2)}$$

where R_1^{CSA} and R_1^{NH} are the longitudinal relaxation rates resulted from the CSA and dipolar interaction, $R_{1\rho}^{CSA}$ and $R_{1\rho}^{NH}$ are the additional dependence on the transverse relaxation rate. Approximating the CSA tensor as an axially symmetric item, these contributions can be expressed as follows：

$$R_1^{CSA} = \frac{3}{4}(\delta_{CSA}\omega_N)^2 J(\omega_N) \quad \text{(S3)}$$

$$R_1^{NH} = \frac{\delta_{NH}^2}{4}(J(\omega_H - \omega_N) + 3J(\omega_N) + 6J(\omega_H + \omega_N)) \quad \text{(S4)}$$

$$R_{1\rho}^{CSA} = \frac{(\delta_{CSA}\omega_N)^2}{4}(\frac{1}{3}J(\omega_1 - 2\omega_r) + \frac{2}{3}J(\omega_1 - \omega_r) + \frac{2}{3}J(\omega_1 + \omega_r) + \frac{1}{3}J(\omega_1 + 2\omega_r)) \quad \text{(S5)}$$

$$R_{1\rho}^{NH} = \frac{\delta_{NH}^2}{4}(3J(\omega_H) + \frac{1}{3}J(\omega_1 - 2\omega_r) + \frac{2}{3}J(\omega_1 - \omega_r) + \frac{2}{3}J(\omega_1 + \omega_r) + \frac{1}{3}J(\omega_1 + 2\omega_r)) \quad \text{(S6)}$$

where ω_1 is the spinlock power expressed in rad/s; ω_H and ω_N are the Larmor frequencies of ^{1}H and ^{15}N expressed in rad/s; ω_r is the MAS rate expressed in rad/s; δ_{NH} is the strength of the N-H dipolar coupling; δ_{CSA} is the chemical shift anisotropy of ^{15}N. $J_{solid}(\omega)$ is the spectral density function in a solid sample：

$$J_{solid}(\omega) = \frac{2}{5}(1 - S^2)\frac{\tau_c}{1+(\omega\tau_c)^2} \quad \text{(S7)}$$

where S^2 is the square of the HN-dipolar order parameter, and τ_c is the effective correlation time. In this work, we adjusted two parameters S^2 and τ_c to make the theoretically calculated $^{15}N\text{-}R_1$ and $^{15}N\text{-}R_{1\rho}$ fitted to the experimentally measured ones simultaneously in Matlab. The quality of the fitting was evaluated with the χ^2, which is expressed as follows:

$$\chi 2 = \left\{ \frac{(R_{1\rho,i,exp} - R_{1\rho,i,cal})^2}{\sigma^2_{1\rho,i,exp}} + \frac{(R_{1,i,exp} - R_{1,i,cal})^2}{\sigma^2_{1,i,exp}} \right\} \quad \text{(S8)}$$

$\sigma_{1\rho}$, σ_1 are experimental errors for $^{15}N\text{-}R_{1\rho}$ and $^{15}N\text{-}R_1$, respectively. The best fit S^2 and τ_c were determined by minimizing the above $\chi 2$ equation. The model calculations were performed based on the Matlab language environment.

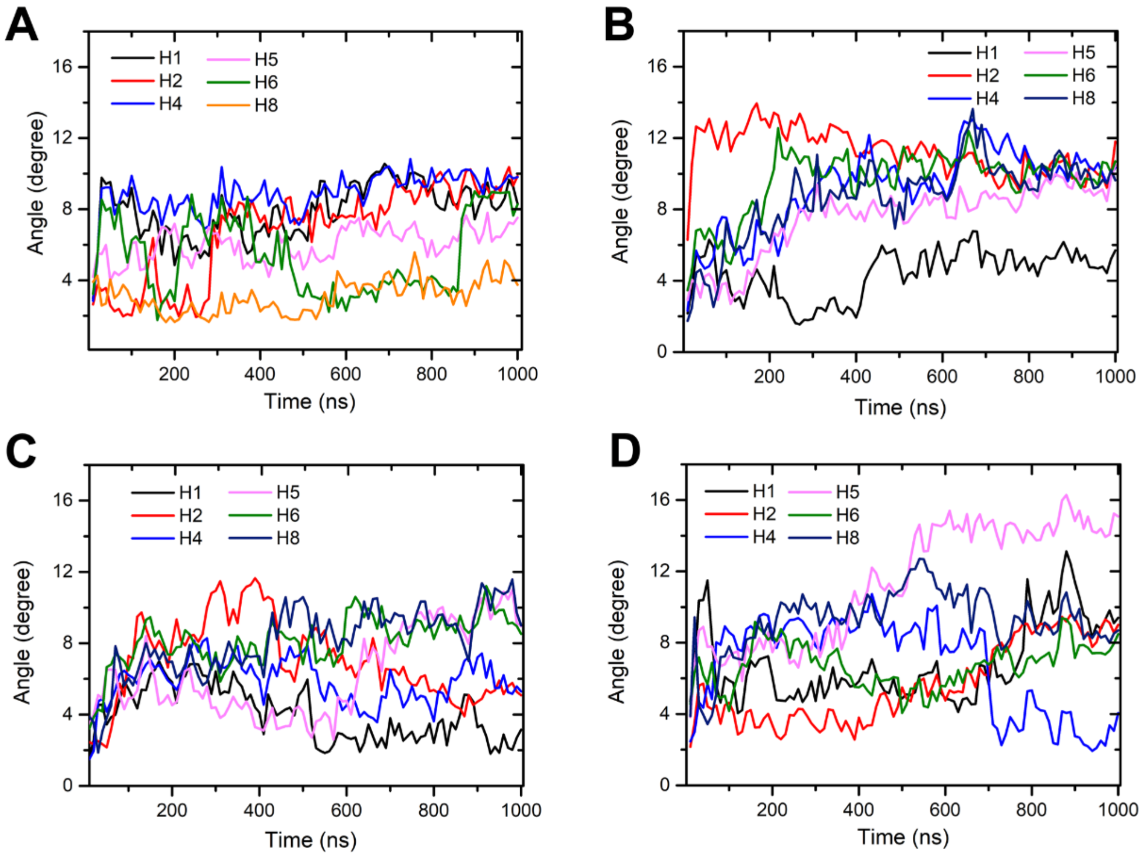

Fig. S10. The fluctuation of orientations of helices in different protomers of AqpZ. (**A**) helices in protomer A; (**B**) helices in protomer B; (**C**) helices in protomer C; (**D**) helices in protomer D. The orientation are calculated as the angles between the helical vectors of structures in the simulations with the initial structure. The helical vectors are defined by the Cα atoms in the two ends of helices.

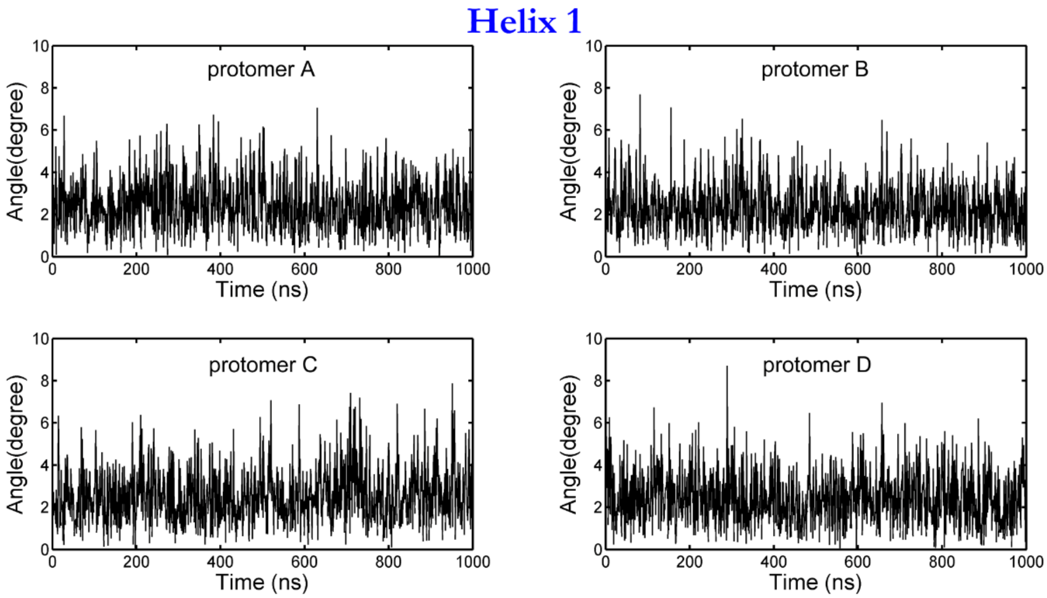

Fig. S11. The orientation angle changes of helices 1 in the structures with 1-ns time interval along the simulation trajectory.

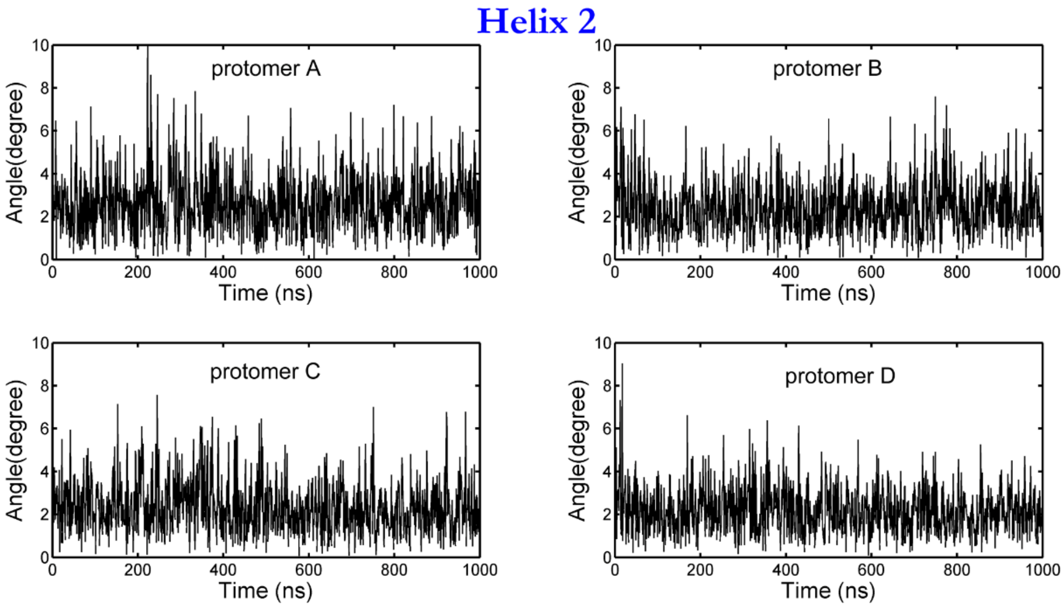

Fig. S12. The orientation angle changes of helices 2 in the structures with 1-ns time interval along the simulation trajectory.

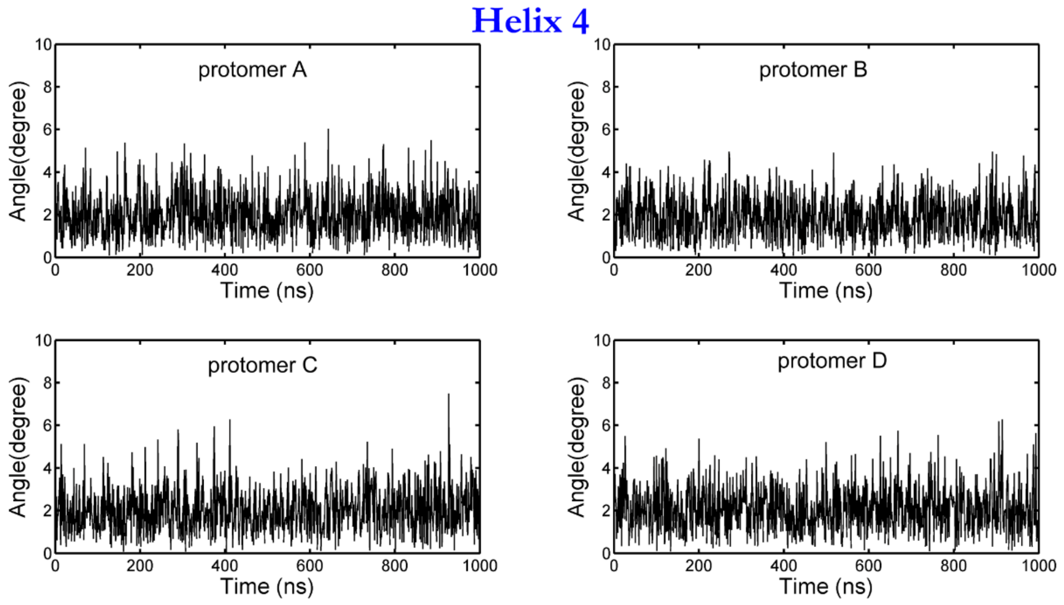

Fig. S13. The orientation angle changes of helices 4 in the structures with 1-ns time interval along the simulation trajectory.

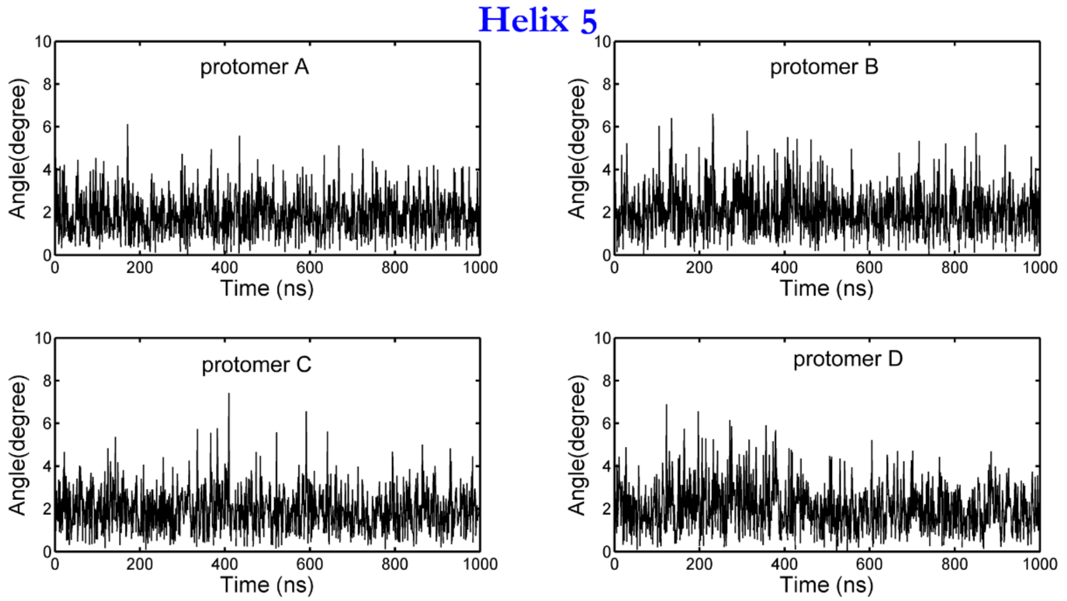

Fig. S14. The orientation angle changes of helices 5 in the structures with 1-ns time interval along the simulation trajectory.

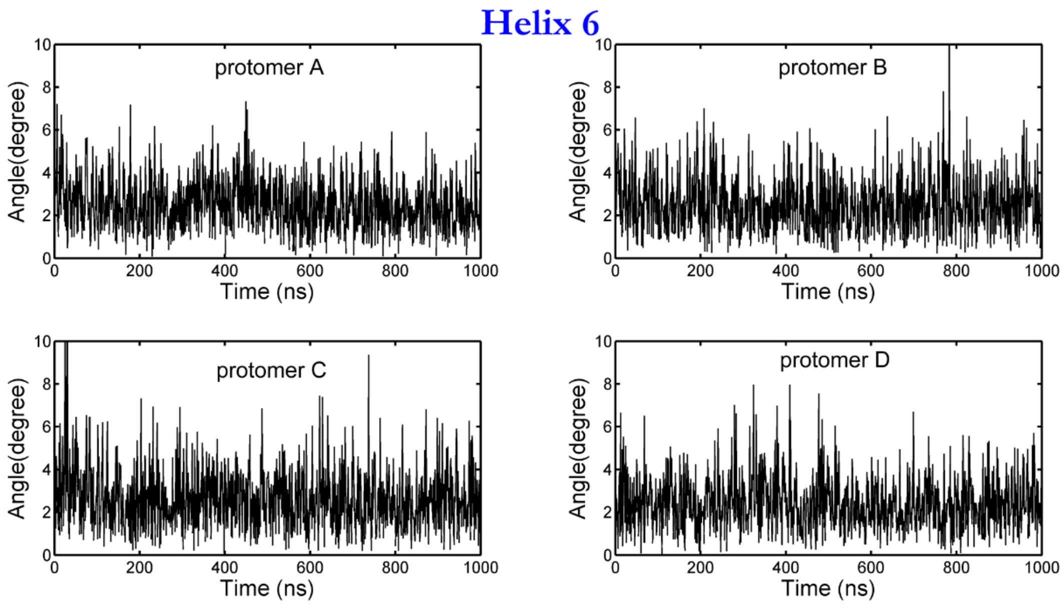

Fig. S15. The orientation angle changes of helices 6 in the structures with 1-ns time interval along the simulation trajectory.

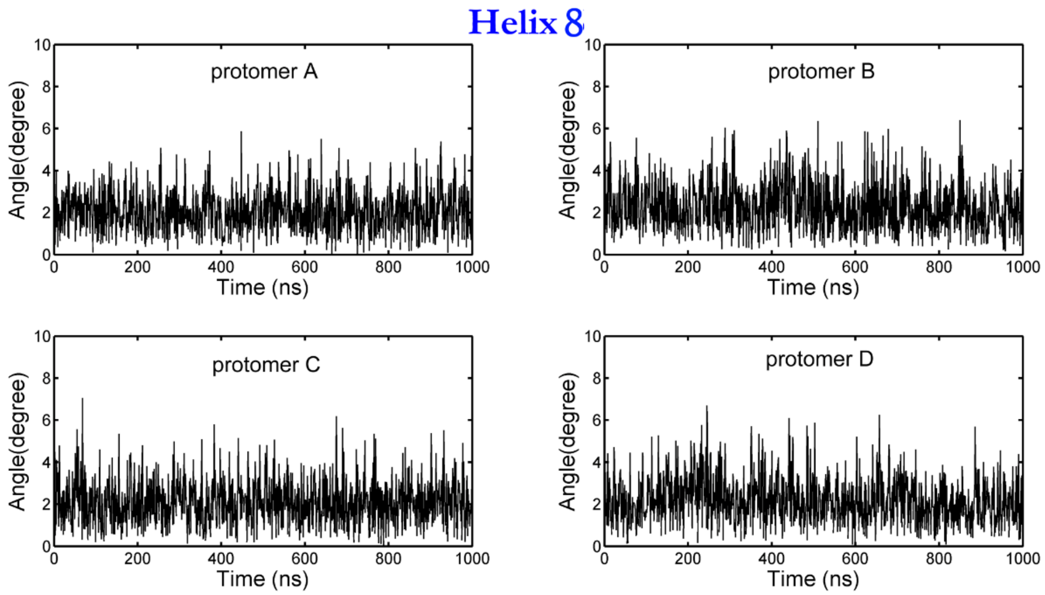

Fig. S16. The orientation changes of helices 8 in the structures with 1-ns time interval along the simulation trajectory.

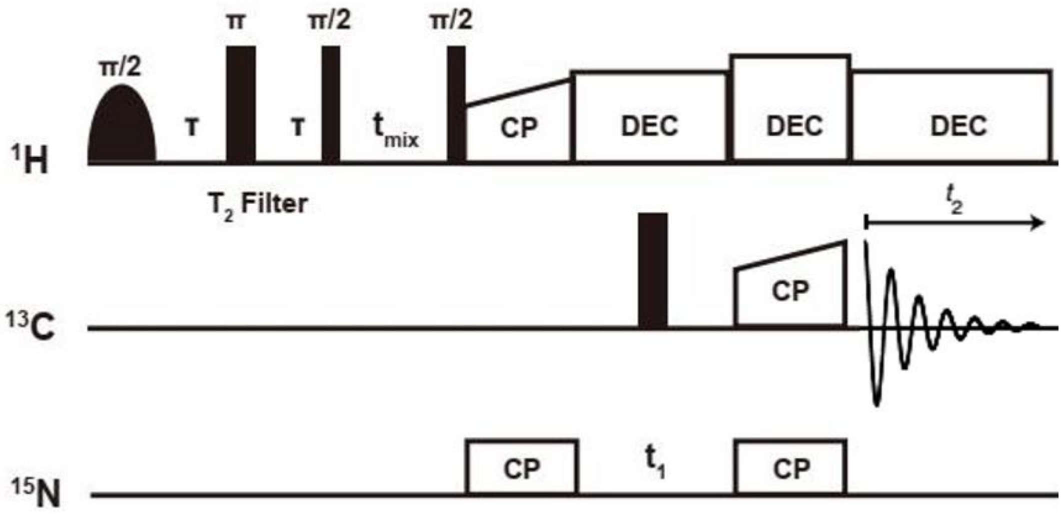

Fig. S17. Pulse sequences of water-edited 2D NCA experiments. A ‘Sinc1.1000’ shape (Bruker TopSpin nomenclature) was chosen to select water ^{1}H signals, 0.9524 ms (10*rotor period) was chosen during T2 filter. ^{1}H-^{15}N CP contact time of 200 μs and short ^{1}H-^{1}H diffusion time of 2.5 ms (t_{mix}) were set to prevent remote ^{1}H spin transfer.

Supplementary Tables

Table S1. Comparison of chemical shifts of uniformly [^{15}N,^{13}C]-AqpZ (*32*) and [10%^{1}H,^{13}C,^{15}N]-AqpZ. The chemical shift differences between these two samples are marked with Δ.

**Residues**	**[^{15}N,^{13}C]-AqpZ (ppm)**			**[10%^{1}H,^{13}C,^{15}N]-AqpZ (ppm)**				**Differences (ppm)**		
	**CA**	**CO**	**N**	**CA**	**CO**	**N**	**^{1}H^{N}**	**ΔCA**	**ΔCO**	**ΔN**
L5	57.5	-	116.2	57.4	179.5	116.0	7.8	0.1		0.2
E8	59.5	179.5	116.2	59.8	179.5	116.4	7.7	-0.3	0.0	-0.2
C9	62.4	177.1	123.6	62.7	177.1	123.6	8.6	-0.3	0.0	0.0
F10	60.2	179.2	116.7	60.6	179.1	116.7	8.8	-0.4	0.1	0.0
G11	47.4	-	107.7	47.7	175.1	107.4	8.9	-0.3		0.3
W14	59.3	174.7	122.0	59.8	174.7	122.0	8.2	-0.5	0.0	0.0
L15	58.3	-	121.4	58.6	179.3	120.8	8.5	-0.3		0.6
F17	62.3	176.9	119.8	62.7	176.9	119.4	9.1	-0.4	0.0	0.4
G18	45.8	175.4	101.3	46.1	175.4	101.3	8.3	-0.3	0.0	0.0
G19	44.9	175.7	111.0	45.2	175.7	111.3	7.9	-0.3	0.0	-0.3
C20	62.2	176.9	118.9	62.5	176.8	119.1	7.4	-0.4	0.1	-0.2
G21	47.8	175.3	107.3	48.1	175.2	107.5	6.8	-0.3	0.1	-0.2
S22	62.7	176.7	118.0	63.0	176.6	118.2	7.2	-0.3	0.1	-0.2
A23	54.9	-	125.4	55.1	179.2	125.7	7.0	-0.2		-0.3
L25	56.0	179.0	114.2	56.3	178.9	114.4	8.5	-0.3	0.1	-0.2
A26	53.2	180.5	117.3	53.5	180.5	117.4	7.3	-0.3	0.0	-0.1
A27	55.9	178.6	125.4	56.2	178.6	125.3	7.9	-0.3	0.0	0.1
G28	44.2	-	102.3	44.5	173.8	102.3	8.8	-0.3		0.0
E31	61.8	178.4	120.9	62.1	178.4	121.4	9.2	-0.3	0.0	-0.5
L32	54.1	177.0	121.1	54.4	177.0	121.1	9.1	-0.3	0.0	0.0
G33	46.8	174.4	103.7	47.1	174.4	103.7	7.6	-0.3	0.0	0.0
I34	63.0	177.1	110.1	63.4	177.0	110.0	7.3	-0.4	0.1	0.1
G35	45.0	172.5	109.4	45.0	172.5	109.1	8.9	0.0	0.0	0.3
F36	56.4	-	117.4	56.3	179.5	117.7	8.6	0.0		-0.3
G38	48.5	-	105.6	48.8	-	105.8	8.1	-0.3		-0.2
A40	55.0	176.3	116.9	55.5	176.4	117.1	7.6	-0.5	-0.1	-0.2
L41	57.5	-	120.6	57.9	176.9	120.8	7.9	-0.4		-0.2
G44	48.3	176.8	104.6	48.6	176.8	104.8	7.2	-0.3	0.0	-0.2
L45	-	181.9	117.3	57.7	181.8	117.3	8.8		0.1	0.0
T46	65.9	175.8	117.1	66.2	175.9	117.3	8.6	-0.3	-0.1	-0.2
V47	66.0	-	123.1	66.3	176.5	123.3	6.7	-0.3		-0.2
T49	65.7	177.4	105.7	66.0	177.4	105.7	8.4	-0.3	0.0	0.0
M50	56.6	178.6	119.5	57.0	178.7	119.7	7.7	-0.3	-0.1	-0.2
A51	55.8	181.5	124.8	56.2	181.5	124.6	9.7	-0.3	0.0	0.2
F52	63.1	177.3	115.9	63.4	177.3	116.4	7.5	-0.3	0.0	-0.5
A53	54.9	177.4	118.2	55.2	177.4	118.4	7.8	-0.3	0.0	-0.2
V54	59.1	178.7	101.2	59.3	178.8	100.4	8.9	-0.2	-0.1	0.8
G55	47.5	-	115.9	47.7	176.3	116.3	8.2	-0.3		-0.4
S58	58.5	176.4	107.0	58.8	176.4	107.2	7.0	-0.3	0.0	-0.2
G59	45.1	174.8	115.0	45.4	174.8	114.9	8.3	-0.3	0.0	0.1
G60	48.0	170.8	110.6	48.3	170.8	110.8	8.0	-0.3	0.0	-0.2
H61	57.0	175.9	123.9	57.2	175.9	123.9	9.6	-0.2	0.0	0.0
F62	54.8	176.6	117.1	55.1	176.6	117.4	11.0	-0.3	0.0	-0.3
N63	51.4	-	107.1	51.7	174.9	107.1	6.4	-0.3		0.0
A65	54.2	180.4	114.0	54.6	180.4	114.0	8.4	-0.4	0.0	0.0
V66	64.3	176.6	118.5	64.7	176.6	118.6	6.9	-0.4	0.0	-0.1
T67	66.6	-	117.3	66.8	176.4	117.6	7.7	-0.2		-0.3
G69	47.8	175.8	110.6	48.1	175.8	110.7	7.8	-0.2	0.0	-0.1
L70	56.8	180.2	122.0	57.1	180.2	121.8	8.5	-0.3	0.0	0.2
W71	60.5	180.3	122.9	60.8	179.8	123.2	8.0	-0.3	0.5	-0.3
A72	54.7	178.0	125.5	55.0	177.9	125.9	8.5	-0.3	0.1	-0.4
G73	45.2	175.7	102.5	45.5	175.4	102.6	8.0	-0.3	0.3	-0.1
G74	45.5	175.3	108.3	45.8	175.3	108.6	7.7	-0.3	0.0	-0.3
F76	55.2	-	123.4	55.6	172.6	123.0	7.6	-0.4		0.4
A78	54.7	180.7	126.3	55.0	180.7	126.7	8.9	-0.3	0.0	-0.4
K79	58.5	-	112.2	58.8	176.9	112.5	8.5	-0.3		-0.3
V81	66.5	-	119.4	66.8	177.4	119.5	7.3	-0.3		-0.1

G83	48.0	175.2	105.5	48.2	175.2	105.5	8.4	-0.2	0.0	0.0
Y84	62.9	-	123.1	63.2	179.2	123.0	7.6	-0.3		0.1
I86	64.7	180.3	118.8	65.0	180.2	119.2	8.2	-0.3	0.1	-0.4
A87	55.9	180.2	124.7	56.4	180.3	124.8	7.8	-0.5	-0.1	-0.1
Q88	58.4	-	116.7	58.7	179.1	117.0	8.3	-0.3		-0.3
G91	49.1	176.5	106.6	49.4	176.4	106.5	8.4	-0.3	0.1	0.1
G92	48.2	-	105.6	48.3	176.6	105.6	8.4	-0.1		0.0
V94	65.3	178.2	124.0	65.6	178.1	124.1	7.5	-0.3	0.1	-0.1
A95	54.8	181.5	120.8	55.1	181.5	121.1	8.6	-0.3	0.0	-0.3
A96	54.9	-	120.9	55.0	178.1	121.0	8.2	-0.1		-0.1
L98	57.3	-	120.2	57.3	176.2	120.6	8.7	0.0		-0.4
Y100	57.6	178.4	120.6	57.9	178.5	120.8	6.9	-0.3	-0.1	-0.2
L101	58.3	179.2	120.7	58.3	179.2	120.7	7.7	-0.1	0.0	0.0
I102	64.5	180.2	118.8	64.9	179.6	119.0	8.2	-0.4	0.6	-0.2
A103	54.7	178.3	124.0	55.0	178.3	124.5	9.2	-0.3	0.0	-0.5
S104	60.3	-	109.1	60.5	173.8	109.5	7.2	-0.2		-0.4
K106	54.2	175.5	121.2	54.5	175.5	122.0	7.0	-0.3	0.0	-0.8
T107	64.7	175.9	123.3	64.8	175.9	123.1	8.3	-0.1	0.0	0.2
G108	45.0	174.5	116.6	45.4	174.4	117.0	9.3	-0.4	0.1	-0.4
F109	59.0	175.0	121.6	59.4	175.0	121.7	7.5	-0.4	0.0	-0.1
D110	52.5	176.2	129.2	52.9	176.1	129.6	7.8	-0.3	0.1	-0.4
A111	55.2	-	131.1	55.5	178.2	131.1	8.6	-0.3		0.0
A113	54.3	179.8	121.0	54.6	179.7	121.2	8.2	-0.3	0.1	-0.2
S114	59.9	176.7	109.0	60.2	176.6	109.2	7.9	-0.2	0.1	-0.2
G115	46.0	173.8	112.0	46.3	173.8	112.1	7.5	-0.3	0.0	-0.1
F116	57.6	172.4	124.5	57.8	172.6	124.5	8.3	-0.3	-0.2	0.0
A117	52.1	178.1	108.5	52.5	178.0	108.7	8.3	-0.4	0.1	-0.2
S118	61.2	175.4	116.7	61.4	175.3	116.7	7.8	-0.3	0.1	0.0
N119	54.6	172.0	122.7	55.0	172.1	122.9	9.2	-0.4	-0.1	-0.2
G120	45.5	170.3	107.5	45.8	170.1	107.6	7.1	-0.3	0.2	-0.1
Y121	54.6	177.2	111.9	54.9	177.3	111.7	8.5	-0.3	-0.1	0.2
G122	46.9	176.6	113.3	47.3	176.6	113.6	8.4	-0.4	0.0	-0.3
E123	57.7	176.6	126.7	57.9	176.6	126.5	9.0	-0.3	0.0	0.2
H124	52.9	173.5	112.5	53.2	173.6	112.8	7.1	-0.3	-0.1	-0.3
S125	54.1	-	114.3	54.5	176.1	114.0	6.7	-0.4		0.3
G127	45.0	173.7	102.0	45.2	173.7	102.2	8.4	-0.2	0.0	-0.2
G128	46.3	175.1	108.5	46.6	175.1	108.5	7.7	-0.3	0.0	0.0
Y129	59.6	176.3	117.4	59.8	176.3	117.4	8.3	-0.2	0.0	0.0
S130	57.3	174.4	115.0	57.5	174.5	115.0	7.9	-0.2	-0.1	0.0
M131	59.6	-	120.5	60.0	177.9	120.6	9.2	-0.3		-0.1
S133	64.5	175.4	116.3	64.7	175.4	116.5	7.8	-0.2	0.0	-0.2
A134	54.6	-	123.2	54.5	177.8	123.2	9.2	0.0		0.0
V136	67.3	177.8	115.5	67.6	177.8	115.8	8.1	-0.3	0.0	-0.3
V137	66.2	-	119.0	66.3	176.9	118.8	8.0	-0.1		0.2
L139	58.9	178.3	122.4	58.9	178.3	122.7	8.2	0.1	0.0	-0.3
V140	66.3	-	115.6	66.5	178.5	115.9	8.2	-0.2		-0.3
S142	63.3	-	116.9	63.6	175.9	117.1	7.9	-0.3		-0.2
G144	47.1	174.8	105.1	47.3	174.6	105.2	7.7	-0.2	0.2	-0.1
F145	58.3	-	121.6	58.3	177.4	121.2	7.5	0.0		0.4
L147	60.2	178.6	126.7	60.4	178.6	127.2	8.3	-0.1	0.0	-0.5
V148	65.6	-	119.6	65.9	177.4	119.6	8.0	-0.3		0.0
H150	61.7	178.6	118.1	62.0	178.6	118.4	8.1	-0.4	0.0	-0.3
G151	48.9	176.1	107.8	49.2	176.0	108.5	8.2	-0.3	0.1	-0.7
A152	53.5	178.1	119.7	53.5	178.0	119.4	8.8	0.0	0.1	0.3
T153	60.4	173.8	102.3	60.6	173.8	102.1	6.9	-0.3	0.0	0.2
K155	58.0	-	129.0	58.3	176.0	128.9	11.0	-0.3		0.1
A157	51.0	-	124.2	51.4	176.4	124.2	7.5	-0.4		0.0
A162	57.0	-	123.6	57.2	175.0	123.8	8.1	-0.2		-0.2
I164	64.1	-	112.8	64.4	176.7	112.2	8.4	-0.3		0.6
A165	55.4	178.4	120.4	55.7	178.4	120.7	7.6	-0.3	0.0	-0.3
I166	66.0	-	115.3	66.1	178.8	115.4	8.8	-0.2		-0.1
L170	57.3	181.6	120.0	57.7	181.5	119.7	7.9	-0.4	0.1	0.3
T171	67.5	-	122.4	67.8	176.1	122.7	8.1	-0.3		-0.3
H174	60.5	-	119.9	60.7	176.3	120.3	7.5	-0.2		-0.4
I176	63.5	175.6	109.9	63.8	175.5	109.9	6.7	-0.3	0.1	0.0
S177	59.6	176.5	100.1	59.9	176.5	99.9	6.6	-0.3	0.0	0.2

I178	66.8	-	129.4	67.1	175.6	129.4	7.5	-0.3		0.0
V180	63.8	179.6	111.3	64.1	179.7	111.0	7.2	-0.3	-0.1	0.3
T181	60.4	177.9	104.9	60.7	177.4	104.9	7.0	-0.2	0.5	0.0
N182	54.0	172.3	124.6	54.4	172.2	124.5	8.8	-0.4	0.1	0.1
S184	-	174.3	128.5	58.7	174.2	129.1	9.2		0.1	-0.6
V185	62.2	175.1	112.2	62.6	175.0	111.7	10.4	-0.3	0.1	0.5
N186	52.0	-	110.7	52.3	174.0	111.0	7.8	-0.3		-0.3
A188	54.8	-	117.0	55.2	178.5	117.0	7.6	-0.4		0.0
S190	62.3	-	114.9	62.6	175.9	115.2	7.8	-0.2		-0.3
T191	66.6	174.1	118.3	66.9	174.2	118.4	8.6	-0.4	-0.1	-0.1
A192	55.8	176.2	118.8	56.2	176.2	119.0	7.3	-0.3	0.0	-0.2
V193	62.3	178.7	97.8	62.6	178.7	97.6	6.1	-0.3	0.0	0.2
A194	54.6	-	124.8	54.6	179.7	125.5	7.2	0.0		-0.7
Q197	60.6	179.6	122.2	60.6	179.6	122.6	8.4	-0.1	0.0	-0.4
G198	46.8	-	98.7	47.1	173.9	98.7	7.6	-0.3		0.0
W200	58.7	177.8	132.4	58.9	177.7	132.3	8.3	-0.3	0.1	0.1
A201	55.8	181.4	124.4	55.8	181.4	124.5	7.0	0.0	0.0	-0.1
L202	56.6	-	115.3	56.9	178.1	115.2	7.1	-0.3		0.1
Q204	56.8	-	113.0	57.1	176.6	112.9	7.7	-0.2		0.1
F207	61.1	175.4	126.8	61.4	175.5	126.8	6.1	-0.3	-0.1	0.0
F208	59.7	173.6	115.6	60.1	173.7	115.7	7.2	-0.4	-0.1	-0.1
W209	60.4	176.9	114.1	60.7	176.9	114.0	7.2	-0.3	0.0	0.1
V210	66.7	176.5	113.6	66.9	176.4	113.7	7.9	-0.2	0.1	-0.1
V211	69.8	-	115.9	70.1	174.6	115.7	7.9	-0.3		0.2
I213	65.7	177.8	112.1	65.9	177.7	112.3	6.7	-0.2	0.1	-0.2
G215	-	175.0	105.6	48.5	175.0	105.7	8.7		0.0	-0.1
G216	47.0	175.4	106.8	47.3	175.4	106.9	7.9	-0.3	0.0	-0.1
I217	65.8	177.3	121.1	66.1	177.2	121.5	8.1	-0.3	0.1	-0.4
I218	66.0	178.0	119.0	66.3	177.9	118.8	8.0	-0.3	0.1	0.2
G219	48.0	176.4	105.6	48.5	176.5	105.7	8.7	-0.5	-0.1	-0.1
G220	47.8	175.1	105.6	48.1	175.1	105.8	8.4	-0.3	0.0	-0.2
L221	57.6	177.6	119.5	57.5	177.5	119.0	8.4	0.1	0.1	0.5
I222	61.6	178.6	122.2	62.0	178.5	122.3	8.7	-0.4	0.1	-0.1
Y223	57.4	-	120.7	57.4	178.4	120.8	7.7	0.0		-0.1
L226	58.2	176.6	123.2	58.2	176.5	123.6	8.1	0.0	0.1	-0.4
L227	57.3	-	117.2	57.4	176.7	117.4	8.6	-0.1		-0.2
K229	59.0	-	122.4	59.4	178.2	122.7	8.2	-0.4		-0.3

Table S2. Experimentally determined $S_{C\alpha H\alpha}$ $S_{NC\alpha}$, ^{15}N-R$_1$ and ^{15}N-R$_{1\rho}$.

Residues	$S_{C\alpha H\alpha}$	σ(±) *	$S_{NC\alpha}$	σ(±) *	^{15}N-R$_{1\rho}$ (s^{-1})	σ(±) (s^{-1})*	^{15}N-R$_1$ (s^{-1})	σ(±) (s^{-1})*
E8	0.94	0.018						
C9	0.93	0.018	0.66	0.11	11.63	1.80	0.003	0.004
F10			0.92	0.09				
G11			0.84	0.03	11.36	1.44	0.002	0.002
L15					4.61	0.15	0.009	0.003
G18			0.90	0.03				
G19			0.93	0.08				
G21			0.82	0.03	5.49	0.49	0.001	0.002
S22	0.93	0.018	0.82	0.04	4.85	0.60	0.000	0.002
A23	0.94	0.018	0.79	0.08				
V24	0.92	0.018						
A26	0.92	0.018	0.87	0.07				
A27					9.52	0.36	0.011	0.001
G28			0.99	0.08	16.95	2.34	0.031	0.007
L32	0.90	0.018	0.88	0.11				
G33			0.88	0.08	13.33	1.07	0.025	0.008
G35					5.65	0.51	0.012	0.005
A37			0.83	0.03				
G38					5.35	0.64	0.002	0.002
A42	0.93	0.018	0.87	0.02				
F43	0.93	0.018	0.86	0.06				
G44					4.59	0.40	0.002	0.004
L45					4.63	0.17	0.017	0.004
T46	0.93	0.018	0.89	0.05				

V47	0.93	0.018	0.92	0.11	6.13	0.57	0.004	0.003
L48	0.93	0.018	0.96	0.07				
T49	0.89	0.018	0.91	0.12				
M50	0.94	0.018	0.82	0.17				
F52					6.29	0.32	0.017	0.006
A53			0.77	0.03	20.41	4.82	0.043	0.012
V54	0.92	0.018	0.91	0.09				
G55			0.81	0.08				
I57	0.95	0.018	0.85	0.05				
S58	0.93	0.018	0.95	0.05				
G59			0.94	0.06				
G60					5.10	0.16	0.006	0.001
H61					4.20	0.21	0.003	0.003
F62					3.60	0.41	0.004	0.003
N63	0.95	0.018	0.88	0.10				
V66	0.95	0.018	0.86	0.06	6.71	0.64	0.002	0.003
T67	0.94	0.018	0.92	0.15				
G69			0.81	0.04				
W71	0.93	0.018	0.85	0.07	4.93	0.32	0.006	0.001
A72	0.92	0.018	0.80	0.05	6.58	0.52	0.007	0.002
G73			0.91	0.08	6.54	0.26	0.007	0.001
G74			0.93	0.06				
R75			0.73	0.12				
F76			0.81	0.04	4.69	0.49	0.000	0.002
A78	0.92	0.018	0.77	0.05				
K79	0.90	0.018			6.41	0.25	0.014	0.003
E80	0.93	0.018	0.87	0.05				
V81			0.90	0.10				
V82	0.93	0.018						
Y84	0.94	0.018	0.82	0.05				
Q88	0.94	0.018	0.93	0.07				
G91			0.87	0.06				
I93	0.96	0.018	0.74	0.18				
V94	0.91	0.018	0.87	0.07				
L98					3.85	0.28	0.011	0.005
Y100	0.94	0.018	0.94	0.10				
I102	0.96	0.018						
A103	0.91	0.02	0.80	0.04	6.45	0.63	0.012	0.007
S104	0.91	0.018	0.91	0.07				
K106			0.72	0.23	7.87	0.50	0.019	0.005
T107	0.88	0.018	0.78	0.18				
G108					10.42	0.43	0.032	0.014
F109	0.91	0.018	0.92	0.06				
D110	0.93	0.018	0.90	0.06	10.31	0.53	0.023	0.006
A111	0.91	0.018	0.80	0.04	9.90	0.69	0.021	0.004
A112	0.94	0.018	0.74	0.06				
A113			0.78	0.10				
S114	0.90	0.018	0.92	0.07	10.00	0.50	0.013	0.002
G115					8.40	0.21	0.008	0.001
F116	0.91	0.018	0.94	0.05	16.67	1.12	0.007	0.001
A117	0.88	0.018	0.91	0.07	4.65	0.30	0.004	0.001
S118	0.94	0.018	0.87	0.03				
N119	0.93	0.018	0.67	0.18	5.88	0.24	0.001	0.001
G120			0.93	0.11	4.85	0.36	0.008	0.001
Y121	0.93	0.018	0.87	0.05				
G122			0.93	0.07				
E123					7.19	0.26	0.019	0.004
H124	0.89	0.018	0.88	0.07	10.53	1.01	0.014	0.004
S125	0.93	0.018	0.84	0.06	6.80	0.23	0.009	0.001
G127			0.95	0.06	4.74	0.20	0.006	0.001
G128			0.90	0.05				
Y129	0.94	0.018						
S130	0.91	0.018	0.92	0.04	8.62	0.45	0.014	0.002
M131					6.71	0.36	0.015	0.002
S133	0.93	0.018	0.87	0.06				
V136	0.93	0.018	0.90	0.06				

V140	0.93	0.018	0.90	0.05				
S142	0.93	0.018	0.82	0.04				
A143	0.94	0.018	0.83	0.03				
G144			0.83	0.06	4.12	0.25	0.013	0.003
F145					12.35	0.61	0.038	0.015
L146	0.91	0.018	0.84	0.07				
L147	0.96	0.018			5.35	0.46	0.003	0.002
V148	0.93	0.018	0.93	0.13	5.43	0.24	0.006	0.001
H150	0.93	0.018	0.82	0.03				
G151			0.88	0.06				
T153	0.89	0.018	0.90	0.36	6.76	0.18	0.004	0.002
D154	0.92	0.018	0.85	0.09	5.35	0.32	0.016	0.004
K155	0.95	0.018	0.96	0.05	6.13	0.30	0.005	0.004
F156	0.94	0.018	0.82	0.05	3.56	0.42	0.005	0.004
A157	0.89	0.018	0.91	0.08	11.76	3.26	0.018	0.003
F161	0.91	0.018	0.96	0.08				
A162					8.77	0.85	0.008	0.001
A165					3.28	0.51	0.009	0.005
I166	0.87	0.018	0.76	0.15	5.05	0.89	0.012	0.001
L170	0.81	0.018						
T171	0.94	0.018	0.92	0.05				
H174	0.94	0.018	0.87	0.05				
I176			0.89	0.06	14.29	2.75	0.021	0.007
S177	0.92	0.018	0.93	0.07	5.26	0.42	0.000	0.003
I178	0.92	0.018			5.32	0.66	0.006	0.003
V180					7.30	1.38	0.013	0.004
T181	0.90	0.018			8.62	0.37	0.002	0.003
N182	0.96	0.018	0.65	0.22	4.27	0.24	0.000	0.002
S184	0.86	0.018	0.88	0.06	10.87	1.69	0.038	0.023
V185	0.92	0.018	0.76	0.08	3.72	0.24	0.006	0.001
N186	0.93	0.018			3.65	0.17	0.004	0.001
A188	0.94	0.018	0.82	0.04				
R189	0.92	0.018	0.94	0.06				
S190	0.93	0.018	0.81	0.03				
T191					6.67	0.31	0.026	0.012
A192	0.91	0.018	0.75	0.10				
V193	0.91	0.018	0.78	0.13	4.90	0.27	0.002	0.002
A194					4.98	0.40	0.002	0.002
I195	0.90	0.018	0.83	0.12				
Q197	0.94	0.018	0.92	0.12				
G198			0.85	0.05	6.29	0.40	0.015	0.002
G199			0.88	0.17				
W200	0.88	0.018	0.93	0.07	7.46	0.79	0.026	0.008
A201					4.81	0.16	0.008	0.001
L202					3.38	0.13	0.011	0.001
E203	0.90	0.018						
Q204	0.90	0.018	0.85	0.14	5.78	0.27	0.012	0.001
F207	0.94	0.018	0.84	0.05	4.76	0.32	0.003	0.001
F208	0.92	0.018	0.93	0.06				
W209	0.94	0.018	0.92	0.11	5.75	0.60	0.010	0.010
V210	0.89	0.018	0.90	0.07				
V211	0.97	0.018	0.84	0.05				
I213	0.90	0.018	0.84	0.32	4.31	0.47	0.000	0.004
V214	0.93	0.018	0.88	0.05	3.68	0.26	0.009	0.008
G216			0.81	0.07	4.93	0.27	0.007	0.004
I217	0.99	0.018	0.89	0.11				
I218	0.93	0.018	0.89	0.06				
G219					4.90	0.24	0.012	0.002
G220			0.82	0.05				
L221					8.06	0.46	0.006	0.005
I222	0.93	0.018	0.84	0.04	6.41	0.29	0.005	0.002
K229	0.93	0.018	0.96	0.07				

* Dipolar lineshape fitting can resolve the differences of ^{1}H-^{13}C dipole coupling with minimum discernible 400Hz (the corresponding errors of order parameters are 0.018). The error of $S_{NC\alpha}$, ^{15}N-R_1 and ^{15}N-$R_{1\rho}$ define a 95% confidence level during formula fitting.

Table S3. Best fitted parameters for anisotropic collective motions of different secondary structural segments of AqpZ using 3D GAF model.

Segments	α(°)	β(°)	γ(°)	θ(°)	φ(°)	τ(ns)	Np*	d.f.*	χ^2-red	AICc*
HELICES 1	12.8	0.0	4.4	16.3	45.2	82.7	15	8	1.8	36.7
HELICES 2	0.0	8.1	13.1	91.8	51.4	55.5	20	13	2.5	50.5
HELICES 3	0.0	7.2	12.0	28.8	18.1	72.5	12	5	0.5	31.4
HELICES 4	13.0	0.0	0.0	57.7	50.1	49.8	18	11	1.3	33.5
HELICES 5	7.7	0.0	12.9	16.7	42.9	69.8	22	15	4.5	84.4
HELICES 6	14.8	0.0	0.0	84.7	58.5	61.4	19	12	5.9	89.3
HELICES 7	1.7	10.6	0.0	38.8	83.6	52.5	18	11	3.7	60.8
HELICES 8	7.5	0.0	12.2	80.3	26.0	54.5	38	31	3.1	109.9

* Np (Np= N1+N2+N3) denotes the number of experimental values used for fittings, where N1, N2, N3 are the numbers of available experimental data for $S^2_{C\alpha H\alpha}$, ^{15}N-$R_{1\rho}$, and ^{15}N-R_1.

* d.f. denotes degrees of freedom, which can be expressed as follows:

$$d.f.= number\ of\ experimental\ values (Np) - number\ of\ fit\ values\ (k) - 1$$

* AICc denotes the Akaike's Information Criterion with correction for the finate sample sizes, which can be expressed as follows:

$$AICc=\chi^2 + 2k(k+1)/(Np-k-1)$$

Evaluation of 3D GAF model fit quality: All transmembrane helices have reduced χ2 values less than 6.0, which are close to 1.0, suggesting that the collective motion of molecular fragments is a good model for explaining most of our data. Compared with the results of ASR reported by Daryl B. Good (*18*), the reduced χ2 values of AqpZ are a little larger. The main reason is that the experimental errors of $S^2_{C\alpha H\alpha}$, ^{15}N-$R_{1\rho}$, and ^{15}N-R_1 of AqpZ are much smaller than that of ASR, so the calculated reduced χ2 values will be larger based on the calculation formula of reduced χ2 (see **Methods**). To evaluate whether the fit is statistically significant, we utilize Akaike's Information Criterion with correction for the finate sample sizes (AICc), which contain much larger penalty for the finate sample size. Compared to the AICc values of ASR based on EMF or 3D GAF analysis (*29*), the AICc values of AqpZ based on 3D GAF analysis are generally smaller, indicating that the 3D GAF fit for AqpZ is statistically more significant.
